# False Memories for Ending of Events

**DOI:** 10.1037/xge0001462

**Published:** 2023-08-31

**Authors:** Petar P. Raykov, Dominika Varga, Chris M. Bird

**Affiliations:** 1 Sussex Neuroscience, School of Psychology, University of Sussex

**Keywords:** false memories, memory distortions, event structure, event endpoints, naturalistic video stimuli

## Abstract

Memories are not perfect recordings of the past and can be subject to systematic biases. Memory distortions are often caused by our experience of what typically happens in a given situation. However, it is unclear whether memory for events is biased by the knowledge that events usually have a predictable structure (a beginning, middle, and an end). Using video clips of everyday situations, we tested how interrupting events at unexpected time points affects memory of how those events ended. In four free recall experiments (1, 2, 4, and 5), we found that interrupting clips just before a salient piece of action was completed, resulted in the false recall of details about how the clip might have ended. We refer to this as “event extension.” On the other hand, interrupting clips just after one scene had ended and a new scene started, resulted in omissions of details about the true ending of the clip (Experiments 4 and 5). We found that these effects were present, albeit attenuated, when testing memory shortly after watching the video clips compared to a week later (Experiments 5a and 5b). The event extension effect was not present when memory was tested with a recognition paradigm (Experiment 3). Overall, we conclude that when people watch videos that violate their expectations of typical event structure, they show a bias to later recall the videos as if they had ended at a predictable event boundary, exhibiting event extension or the omission of details depending on where the original video was interrupted.

## Public Significance Statement

Our memory for events is fallible: We often forget or distort certain details or even remember things that never actually happened. Here, we showed that participants often inaccurately remember how events ended if the events were interrupted unexpectedly. For instance, if a video clip of a cyclist preparing to ride a bike was interrupted before the cyclist left, people would often falsely remember that they saw the person riding their bike away at the end. Conversely, if the video clip continued to briefly show the cyclist arriving at their destination before the clip was finished, participants would often not recall this additional detail and remember that the clip ended with the person preparing to ride their bike. These experiments highlight how people are biased to recall events as having well-defined end-points, even when that conflicts with what was actually experienced.

We can usually remember what we ate for breakfast this morning, but we are unlikely to have a veridical memory for everything that happened to us since we woke up. Decades of research have shown that memories are not perfect recordings of the past, they are prone to errors and biases (Johnson et al., 1993; Loftus & Pickrell, 1995; Schacter, 1999, 2022). Our world knowledge, in the form of “schemas” and “scripts,” as well as our beliefs and attitudes, influence how we experience and later remember events (Alba & Hasher, 1983; Bransford & Johnson, 1972; Dooling & Christiaansen, 1977; Schacter et al., 2007; Schank & Abelson, 1977). Nevertheless, rather than reflecting a dysfunctional memory system, the interaction of prior knowledge and episode-specific details are likely to serve as an adaptive process—enabling us to “fill in” information about incomplete memories (Hemmer & Steyvers, 2009; Huttenlocher et al., 1991) and also to “compress” memories of detailed events (Nagy et al., 2020). This raises an important question: to what extent are our memories of events driven by what actually happened, versus our expectations of what usually happens? Here, we address this question by investigating the conditions that make us susceptible to misremembering the endings of events.

We tend to chunk ongoing experience into discrete events (Zacks & Tversky, 2001). A mental representation of the current, ongoing, event (an “event model”) is held in working memory and helps predict incoming sensory information (Radvansky & Zacks, 2017). When the current event model is no longer useful in predicting what will happen next, an “event boundary” is triggered and a new event model must be created. Each event corresponds to a unit of experience that is coherent in itself. Event boundaries are particularly informative about the events that have just taken place (Newtson et al., 1977; Zacks et al., 2001), and they tend to be better remembered and better at capturing attention than information in the middle of events (see for review Zacks, 2020). Endpoints tend to be detected when there is a change in an element that is central to that particular event (e.g., location, the people involved, their goals, etc.). In particular, the completion of action goals is a defining point in our stream of ongoing behavior, and is consistently identified as an event boundary (Magliano et al., 2014; Zacks, 2020; Zwaan & Radvansky, 1998). Furthermore, people are able to anticipate when an event is expected to end (Baldwin & Baird, 2001; Baldwin & Kosie, 2021; Shin & DuBrow, 2021).

People sometimes also falsely fill in information about what caused an event simply because they saw a result that implied a specific cause. For example, Strickland and Keil (2011) found that participants who viewed a video clip of a person running toward a football, followed by a movie shot showing the football flying away, were more likely to falsely recognize seeing the person kicking the ball compared to those who did not see the outcome of the kick. Interestingly, false recognition of inferred actions is found even a few seconds after watching the clips, suggesting that these actions are encoded as part of the overall event (Papenmeier et al., 2019). Similarly, Hannigan and Tippens Reinitz (2001) showed participants sequences of photos depicting everyday situations and found that they falsely recognized unseen casual actions as having been presented, when, in reality, only the outcome of the action was presented at encoding. Thus, event endings may bias the retrieval of the middle of the event in order to create a coherent memory.

What about memory for events that are missing a coherent ending? The studies above revealed that participants falsely recognize implied actions from the middle of events, but they did not find evidence for false recognition of actions from the end of an event when these were omitted during the initial presentation (Hannigan & Tippens Reinitz, 2001; Papenmeier et al., 2019). Nonetheless, there is other evidence to suggest that people do activate information about an anticipated ending when remembering incomplete events. Frisoni et al. (2021, 2022) showed that participants’ judgment of when events took place on a movie’s timeline is affected by the presence of the final part of the movie. When the final part of the movie was cut out, participants were more likely to underestimate the timing of scenes close to the interrupted end point compared to when the ending was intact. This suggests that participants could have represented the anticipated conclusion of the movie, and this biased their estimation of the location of scenes within the timeline of the movie. This indicates that not only gaps in the middle of events may be filled in memory, but missing endings may also be represented.

In the bulk of the studies considered so far, gaps in memory were filled in on the basis of schema, script knowledge or through familiarity with the type of situations depicted. Nevertheless, there is also evidence that people have a more abstract knowledge of the typical structure of events—that they have recognizable beginnings, middles, and ends. For example, the fact that people are so remarkably consistent in their placing of event boundaries demonstrates that there is a generic understanding of what an event is (Shipley & Zacks, 2008). Interestingly, people can also intuit whether an ongoing event is likely to have a well-defined ending and are able to distinguish between so-called bounded and unbounded events even if they depict very different situations (Ji & Papafragou, 2020).

There has been little research on how event structure and event boundaries may shape the recall of naturalistic events. It has been argued that event segmentation during encoding creates an event unit that is later recalled as such (Ezzyat & Davachi, 2011). The idea of an event as a discrete unit of memory is consistent with the observation that location–object–person triplets that were imagined together at encoding tend to be retrieved or forgotten in an all-or-none fashion (Joensen et al., 2020). Similarly, when recalling a complete movie, whole scenes tend to be recalled or not, but are occasionally recalled in the wrong temporal sequence (Chen et al., 2017). However, in contrast to the concept of an event as a fixed unit, it has been suggested that event boundaries can change through the act of retrieval, and by doing so, change the very definitions (the beginning and end) of events in memory (Zacks, 2020). Consistent with this, Hohman et al. (2013) showed that the boundaries of older autobiographical memories are often extended by including additional details into the remembered episode that were initially considered outside of the event’s boundaries. This indicates that what we remember as cohesive units of experience do not necessarily depend only on segmentation processes at encoding, but the event boundaries can be redefined at retrieval.

## The Present Experiments

**Figure 1 fig1:**
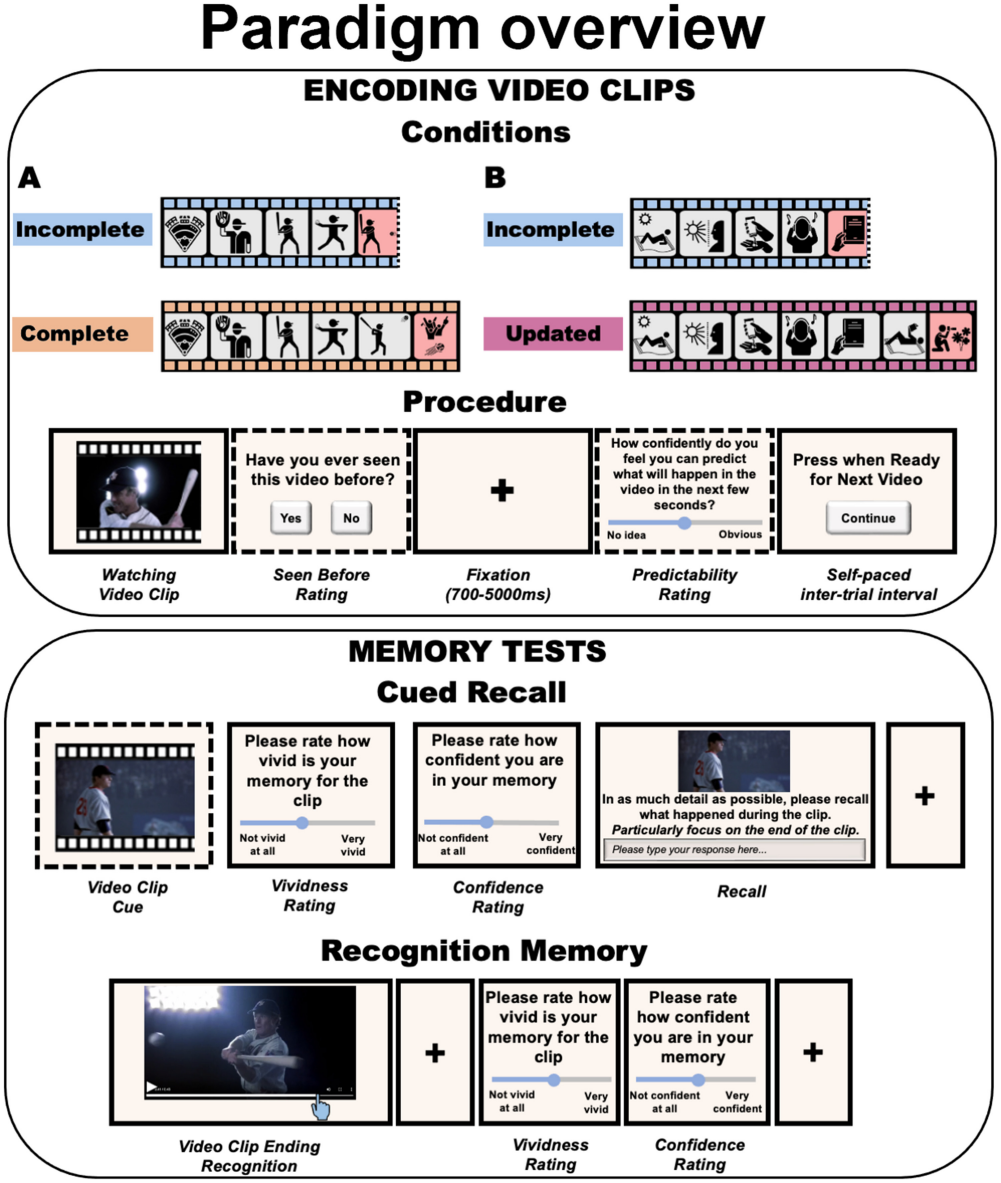
Design *Note.* Encoding phase (top panel): participants watched 24 short videos. In Experiments 1–3, (A) videos were stopped either just before an action was completed (incomplete condition); or shortly after the action was completed within the same context (complete condition). In Experiments 4 and 5, (B) video clips were stop-motion videos and were stopped either just before an action was completed (incomplete condition); or shortly after the start of a new scene (updated condition). The procedure during encoding was nearly identical in all experiments, except that some of them did not include a predictability rating (Experiments 2, 4, 5); memory test phase (bottom panel): In Experiments 1, 2, 4, and 5, participants described what happened in the videos, especially focusing on the end of the clips (cued recall). On each trial, they saw a cue (a few seconds of the beginning of the clip—Experiments 1 and 2; or a blurred screenshot of the first frame—Experiments 4 and 5), provided vividness and confidence ratings before their description. In Experiment 3, participants watched a longer version of the video clips and stopped the clip at the point when they judged the original video had ended (recognition memory). Adapted from *thenounproject.com*, by Icons and Photos For Everything, 2023 (https://thenounproject.com/). In the public domain. See the online article for the color version of this figure.

To summarize, people process and remember their experiences as coherent events, and they broadly differentiate between events that have well-defined endings and those that do not. Furthermore, we have seen that event boundaries play a privileged role in memory, and that missing endings to movies can influence time estimation judgments as if they had occurred. Moreover, inferred but unseen, details from an event can be represented as if they had occurred, and also event boundaries can be redefined at retrieval as well as during encoding. In the present study, we investigate how people remember videos that violate the normal experience of events that finish at a coherent event boundary (see Figure 1 for an overview). Given the importance of event boundaries for comprehending situations, and the fact that people falsely recognize inferred elements of events that were never actually seen, we expect that people will show a bias to retrieve completed events, even if this was not what was presented. If a video is curtailed, ending before a meaningful piece of action, then people may later falsely recall an inferred ending to the video, extending the remembered event beyond what was actually watched (incomplete condition). Conversely, if the video ends by showing the beginning of a new scene, then at recall, this additional scene may be omitted from recall, effectively “trimming back” the remembered video to a salient event boundary (updated condition).[Fig fig1]

An opposing prediction to consider is that violating people’s expectations of when the videos will end induces a prediction error that actually improves memory for the ending. Prediction error often leads to better learning (Quent et al., 2021; Stawarczyk et al., 2020), and surprising actions are recognized more accurately than actions that are consistent with a particular situation (Ben-Yakov et al., 2021). Nevertheless, the relationship between prediction error and memory performance is complex; in some studies, both particularly high and low prediction error can benefit memory, while intermediate levels confer no advantage (Greve et al., 2019), yet in other studies, the opposite pattern is found (Ortiz-Tudela et al., 2023). In addition, there is a large literature showing that in many situations, events that are congruent with prior knowledge are better remembered (Bein et al., 2014; Brod & Shing, 2019; Craik & Tulving, 1975; Gilboa & Marlatte, 2017; Ortiz-Tudela et al., 2017; Raykov et al., 2021). Furthermore, there has been very little research on the effect of omitting an expected stimulus on its subsequent memory. Therefore, even though the videos in our incomplete and updated conditions are likely to elicit prediction error, we nevertheless hypothesize that memory for the endings of these videos will be more prone to error than those in the complete condition.

Most of our experiments used a retention interval of one week. This was to avoid ceiling effects and to be more sensitive to memory errors, since longer delays between study and retrieval tend to increase memory distortions, such as participants remembering events to be more in line with their prior knowledge and beliefs (Bartlett, 1932; Bergman & Roediger, 1999; McClelland et al., 1995; D. A. Smith & Graesser, 1981; Winocur et al., 2010). However, we also tested memory in the same session as encoding (Experiment 5), to compare memory recall at short retention intervals compared to the longer intervals used in the other studies. If false memory recall was disproportionately seen at the longer test delays, this would implicate memory reconstruction processes operating during retrieval. However, if the video endings were falsely recalled even after short retention intervals, then it would suggest that alternate endings might have been encoded when the videos were watched.

All experiments except one used a cued recall paradigm to test memory (Experiment 3 used a recognition paradigm). By contrast, previous studies of how inferences affect memory for events have tended to use recognition paradigms (Hannigan & Tippens Reinitz, 2001; Papenmeier et al., 2019). However, in everyday situations, we frequently need to recollect information about events; it is, therefore, important to establish how much our recall is based on prior knowledge. Furthermore, free recall arguably provides a more direct measure of memory representations that are consciously accessible (Gelbard-Sagiv et al., 2008; Yonelinas, 2002). By additionally testing recognition memory for the videos, we can establish whether any effects generalize across all retrieval situations or are specific to some. For example, if false memories arise as a result of retrieval processes, then the additional cuing afforded by a recognition paradigm should enable people to access an accurate representation of the event, even if this representation was not accessible at the point of recall.

## Experiments Procedure Summary

A schematic of the general paradigm used across all of the experiments is shown in Figure 1.

All experiments were run online using jsPsych (De Leeuw, 2015) using a privately hosted web-server-cPanel. Participants completed the experiments without direct experimental supervision. In each experiment, participants first watched 24 (12 incomplete) short (∼35 s) video clips at their own pace and their memories were tested afterwards. In Experiments 1, 2, 3, and 5a, we used video clips sourced from the internet, in Experiments 4 and 5b, we used custom-made video clips. For a selection of the video clips in different conditions, see https://tinyurl.com/a5epxzab. In Experiments 1–4, we tested participants’ memory 1 week after they had studied the clips. In Experiment 5, we tested participants’ memory for the clips immediately, after they watched all 24 video clips. This meant there was still a short delay between encoding the clips and remembering them. In Experiment 3, we tested participants’ memory with a recognition memory paradigm, whereas in all other experiments, we used a cued recall paradigm. In all experiments, memory for the videos was tested in random order. We collected predictability ratings about the clips during the encoding session for Experiments 1, 3, and 6 (see the online supplemental materials).

### Transparency and Openness

Hypotheses were preregistered for Experiments 2 (https://osf.io/69k3s), 4 (https://osf.io/fuyva), and 5 (https://osf.io/ybp52). Data and analysis code are available at https://osf.io/s3d5z/.

## Experiment 1

The main aim of Experiment 1 was to test how interrupting video clips before, or at, a natural endpoint would affect memory a week later for the ending of the clips. We were particularly interested if memory is biased toward recalling coherent, completed events, even if the viewed event stopped before its typical conclusion. We hypothesized that when situations are interrupted before their typical ending (incomplete condition), participants would be more likely to falsely recall details beyond the interrupted point compared to when situations are interrupted at their expected conclusion (complete condition). To measure false memory errors, we scored an “extension memory error” for each video clip where a participant recalled any additional details (that were never shown) about the ending of the clip.

### Method

#### Participants

Participants were recruited from the undergraduate psychology course at the University of Sussex, provided informed consent, and completed the experiment in exchange for course credits. There were no additional exclusion criteria for participating. The research project was approved by the University of Sussex Cross School Research Ethics Committee. The study was open for recruitment for 8 weeks, during which time 223 participants completed the encoding part of the experiment. From this sample, only 89 completed the recall phase of the experiment. The high dropout rate may have been because participants were only rewarded with course credits. Of the 89 participants, two were excluded since they had watched the encoded videos twice. This sample size provides 80% power to detect a small to medium effect size (Cohen’s *d* = 0.30: G*Power calculation for a paired *t* test).

Demographic information for all experiments was self-reported. Participants were asked to type in their gender, and year of birth. The final group of 87 participants (10 male) had a *M*_age_ of 19.9 years (*SD* = 1.95 years).

#### Materials

Stimuli were 24 unrelated video clips sourced from films and television shows available online. All video clips showed meaningful storylines that involved different characters and activities (e.g., diving and office activities). The average length of the full video clips was 40.64 s (*SD* = 9 s; range 22.46–55.42 s).

We created two versions of each of the video clips. The “complete” version showed the full video clip ending after the completion of an action. The “incomplete” version showed the video clip ending before the final action was completed. For instance, one video clip showed a pitcher throwing a baseball to a batter. The complete version ended after the batter successfully hit the ball and the camera showed the reaction of the audience. The incomplete version ended before the batter hit the ball (see https://tinyurl.com/a5epxzab). The incomplete versions were on average 6.48 s (*SD* = 1.83 s) shorter than the complete versions. We created two counterbalancing lists across participants, in which we varied which video clips were in the incomplete and complete conditions. Half (12) of the video clips in a list were the complete version and the other half (12) of the video clips were the incomplete version.

#### Procedure

Participants performed both the study and memory session online. In Session 1 (encoding phase), participants watched all 24 video clips in a random order (12 from the complete condition and 12 from the incomplete condition). Participants were assigned randomly to one of the two counterbalancing lists before the experiment started.

Participants were instructed that they would watch 24 video clips and their memory for the clips will be tested a week later. They were not informed that some of the video clips would end abruptly. Because videos were taken from materials that are widely available online, we included a question after each video to ask if the participant has seen the video before (referred to as “SeenBefore” in analyses below). After watching each video, participants were presented with a fixation cross for a randomly jittered duration between 700 and 5,000 ms. Participants also provided a rating from 0 to 100 how confidently they feel they can predict what will happen in the video in the next few seconds (predictability rating). This allowed us to examine subjective predictability ratings for each video. It has been suggested that participants are much better at predicting what would happen next within an event than across event boundaries (Eisenberg et al., 2018; Franklin et al., 2020; Zacks et al., 2011). Participants had to press a button indicating that they are ready to watch the next video. As such, this was a self-paced study task. If a participant clicked on another webpage in the browser, they were shown an alert message asking them to stay on the experimental page for the whole duration of the experiment and blurring the nonexperimental web pages.

One week after completing the Encoding Phase, participants were emailed the link to complete Session 2 (recall phase). During the recall phase, participants saw the first few seconds of each video and answered questions regarding their memory for the clip. Participants’ memory for the videos was tested in a random order. The average cue duration was 5.40 s (*SD* = 0.35 s; range = 5.03–6.4 s). After each memory cue, participants were asked to rate how vivid their memory for the clip was on a scale from 0 to 100. Participants also provided memory confidence ratings for each video. Participants were then asked to describe in as much detail what they recall happened during the clip. Participants were asked to focus particularly on the end of the clip. Participants entered their responses in a text box in a self-paced manner. After entering their response for a video, participants saw a fixation cross for randomly jittered duration between 700 and 2,500 ms.

#### Data Scoring

Data from all 87 participants who completed the recall phase were analyzed. Responses were rated by one of the authors (Petar P. Raykov). We rated all responses that provided correct details about the video that could not be seen during the cue as remembered. From these ratings, we categorized each trial as being remembered or forgotten. For instance, one participant wrote: “The badgers cuddle and a man comes on the screen to explain what they are doing.” Although this was correct it described only the first 5 s of the video they saw as a memory cue and therefore was labeled as forgotten. Apart from giving a *remembered/forgotten* score for each video, we also rated whether participants reported additional details of the video endings that were not shown. We label these as “extension” errors as they result in a recalled event that is an extended version of what was actually watched. Extension errors were scored as *present* (1) or *absent* (0).

An extension error was scored as being present on trials where a participant recalled *any* additional detail that was a plausible continuation of the video clip. This included, but was not limited to, recalling details beyond an interrupted action at the end. For example, in one video in the incomplete condition, a boy is shown jumping onto ice and then stops. A response that was scored as an extension error was: “The boy jumps on the ice and it breaks into separate pieces. He tries to stay above the water but eventually ends up falling in.” Note that the number of additional details recalled was not counted; extension errors were scored as *present* (1) or *absent* (0). Conversely, responses that specifically stated that the boy did not fall or did not mention the boy falling into the water were given a score of 0 (e.g., “The people around him encourage him to jump so he starts jumping and he gets his shoes wet” [note that some water splashes were visible before the video clip ended]). The video clips used in the complete condition were all taken from longer narrative movies, so although they ended at event boundaries, it was still possible to infer additional details about what was likely to happen next. Consequently, extension errors were also found for these videos. An example extension error in the complete condition was after watching a baseball match and recalling that the batter “hits the ball and the crowd goes wild as he runs” at the end, when the video clip ended with the batter hitting the ball and the audience looking surprised, but never showing the batter starting to run across the bases.

Occasionally, participants would falsely recall details from other parts of the videos than the end. For example, in one clip, an old man sets a mousetrap and multiple participants later intruded that the mouse was trying to get the cheese (no cheese was shown in the video). These memory intrusions were not scored as extension errors and were not analyzed here.

#### Data Analysis

Data from all experiments were analyzed using logistic mixed effect models estimated with the lme4 (Bates et al., 2014) package available in R. Figures were created using Matplotlib and Seaborn libraries in Python.

Our main interest was whether participants would make more extension errors when remembering the videos from the incomplete condition. Therefore, we focused only on trials that were remembered. We excluded five participants who recalled fewer than 40% of all videos (10 out of 24). We note including these participants does not affect our main conclusions. To examine whether the conditions affected the proportion of extension memory errors, we ran a mixed-effect logistic regression. We entered the binary extension score as the dependent variable and included a predictor indexing whether the video was encoded under the *incomplete* (0) or *complete* (1) condition, a binary predictor of no interest indicating whether participants had seen the video before, random intercepts for participant and video, and random slope for condition across participants [Extension ∼ Condition + SeenBefore + (Condition | Subject) + (1 | Video)].

Additionally, we examined whether overall memory performance and subjective ratings differed between conditions using similar models—for example, [RememberForgotten ∼ Condition + SeenBefore + (Condition | Subject) + (1 | Video)]. We also examined whether videos seen in the incomplete conditions were perceived as having more predictable continuation compared to videos in the complete condition.

### Results

**Figure 2 fig2:**
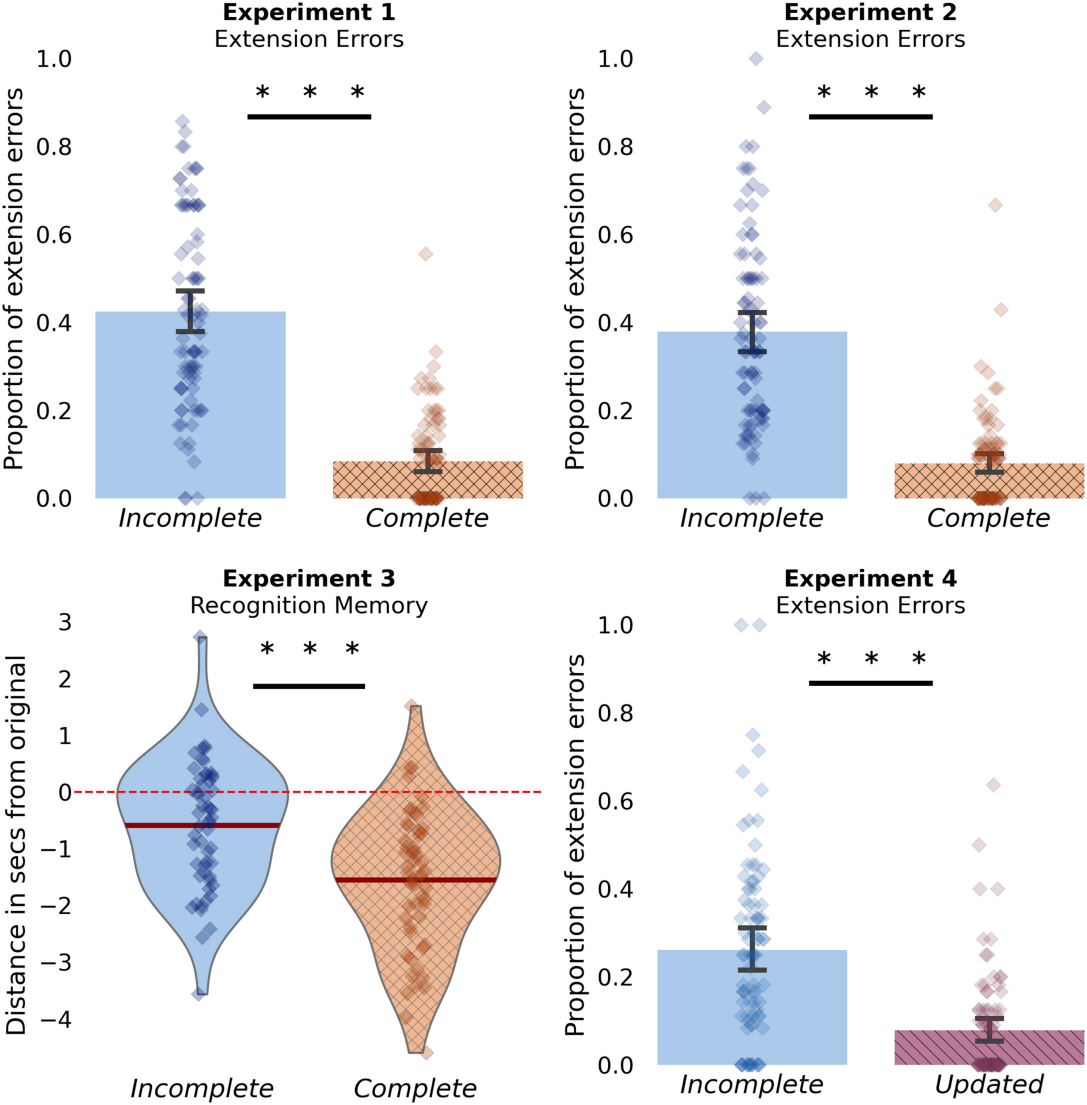
Summary Figure for the Main Results in Experiments 1–4 *Note.* Across the free recall paradigms in Experiments 1, 2, and 4, participants were more likely to recall additional details about the ending of clips (extension errors) in the incomplete condition compared to the complete and updated conditions. In the recognition memory paradigm in Experiment 3, similar false memory errors about the ending were observed less robustly, instead, participants were more likely to underestimate when the clips ended in the complete condition compared to the incomplete condition. See the online article for the color version of this figure.

Participants on average remembered 76.5% of the clips during the cued recall task. Participants made extension errors on 24.9% of all remembered trials. Furthermore, we observed that participants made more extension errors for remembered videos in the incomplete condition when compared to the videos in the complete condition (42.5% vs. 8.2%; β = −2.43, 95% CI [−2.89, −1.97]; *Z* = −10.31; *p* < .001; see Figure 2). This is in line with our initial hypothesis that participants will be more likely to falsely add information during retrieval of abruptly interrupted video clips.[Fig fig2]

During the study session, we asked participants to rate how confidently they could predict what would happen next for both the incomplete and complete videos. We found that predictability ratings were higher for incomplete versus the complete videos (72.34 ± 25.47 vs. 56.48 ± 29.84; β = −15.1; 95% CI [−17.47, −12.64]; *t*_73.20_ = −12.22; *p* < .001; see Figure S1 in the online supplemental materials). This is in line with previous research showing that participants are better at predicting what will happen within events than across events (Eisenberg et al., 2018; Zacks et al., 2011). This was true also when we focused only on the examining the predictability ratings in the later remembered videos (incomplete vs. complete, 75.75 ± 22.93 vs. 57.90 ± 29.97; β = −16.29; 95% CI [−18.87, −13.71]; *t*_872.35_ = −12.38; *p* < .001).

Participants also showed slightly worse overall memory for the clips in the incomplete condition compared to the complete condition (74.6% vs. 78.5%; β = 0.32; 95% CI [0.01, 0.63]; *Z* = 2; *p* = .04) and reported lower vividness (62.26 ± 25.95 vs. 66.33 ± 25.24; β = 5.21; [2.7, 7.73]; *t*_75.06_ = 4.07; *p* < .001) and confidence (59.94 ± 26.29 vs. 63.65 ± 26.4; β = 4.65; [2.07, 7.23]; *t*_75.54_ = 3.53; *p* < .001) for the incomplete versus the complete condition, when focusing on the remembered trials.

In the online supplemental materials, we report a post hoc analysis where we examined whether the predictability ratings during encoding were associated with the extension errors. Specifically, we were interested in testing whether participants made more extension memory errors for the videos for which they could more confidently predict what would happen next. Interestingly, we found evidence that the predictability ratings were not associated with making extension errors (predictability, β = −0.01; null model does not include predictability, BF_01_ = 9.37; see the online supplemental materials).

## Experiment 2

In Experiment 1, we observed that participants made more extension errors for the incomplete videos compared to the complete videos. We ran a second experiment that closely followed the procedure of Experiment 1. The main difference was that for Experiment 2 we removed the predictability rating that followed each video during encoding. We reasoned that being asked to predict what will happen in the next few seconds after each video could have influenced how the video clips were encoded. It is possible that participants imagined a possible ending during the predictability rating which could have led to a source confusion during retrieval. Specifically, because participants rated the incomplete videos as having more predictable continuation compared to the complete videos, we wanted to ensure we would observe the extension errors even if participants did not have to actively try and predict how the video would end during encoding. This experiment was preregistered before any data collection was initiated (https://osf.io/69k3s).

### Method

#### Participants

Participants completed both tasks online and were recruited from Prolific (https://www.prolific.co/). We recruited initially 97 (62 female and 35 male) participants who completed both sessions. The *M*_age_ of participants was 26.85 ± 5.11. We aimed to recruit at least a sample size of 80 usable datasets, which would have allowed us to achieve 99% power to observe a medium-sized effect. Note in Experiment 1, we observed a condition difference in the extension errors of −2.2 logOdds, which translates to Cohen’s *d* of −1.2 or a large effect size (Sánchez-Meca et al., 2003). We excluded 11 participants who recalled fewer than 40% of all the videos (10 out of 24). Including these participants in the analysis does not change our main results. This left us with 86 participants as a final sample size. Participants were compensated with £8 for completing both sessions. The research project was approved by the University of Sussex Cross School Research Ethics Committee.

#### Stimuli and Procedure

The same stimuli were used here as in Experiment 2. The procedure was near identical to Experiment 1. The only difference was that here participants did not make any predictability ratings during the encoding session.

#### Data Scoring and Data Analysis

Data scoring procedure for Experiment 2 was identical to Experiment 1 described above. Responses were rated by one of the authors (Petar P. Raykov). Our main interest was in the extension errors and therefore we focused on recalled trials. The logistic mixed effect regression model had the same parameters as in Experiment 1 [Extension ∼ Condition + SeenBefore + (Condition | Subject) + (1 | Video)].

In addition to the extension errors, we also examined differences in Remembered/Forgotten responses across conditions, and differences in participants’ subjective memory ratings.

### Results

Participants in Experiment 2 overall remembered 69.85% of the clips in the cued recall task. Participants made extension errors on 21% of the remembered trials. We replicated the effect we found in Experiment 1 observing that participants are much more likely to make an extension memory error for incomplete rather than complete video clips (36.4% vs. 7.6%; β = −2.12; 95% CI [−2.45, −1.78]; *Z* = −12.38; *p* < .001; see Figure 2). Due to convergence issues, we did not include a random slope for the effect of condition. Therefore, as preregistered we fitted a simplified model that included a random intercept for participant and video [Extension ∼ Condition + SeenBefore + (1 | Subject) + (1 | Video)]. These results replicate Experiment 1 and demonstrate that the previously reported effect was likely not observed only because participants had to judge each video’s predictability after they watched it.

Similar to Experiment 1, participants showed worse memory for the incomplete versus complete condition (65% vs. 74.6%; β = 0.67; 95% CI [0.45, 0.90]; *Z* = 5.87; *p* < .001). Due to convergence issues, we had to drop the random slope effect, fitting the model as [RememberForgotten ∼ Condition + SeenBefore + (1 | Subject) + (1 | Video)]. Interestingly, previous work has shown that predictable, and completed events are better remembered (Boltz, 1992; Gold et al., 2017; Schwan & Garsoffky, 2004). Boltz (1992) asked participants to watch a movie and inserted commercial breaks at natural or unnatural points during the movie. They found that participants had worse memory for the movie with breaks presented at unnatural event boundaries in comparison to commercial breaks that accented the natural structure of the movie. Our findings of worse recall for incomplete versus complete events agree with Boltz’s results.

Replicating Experiment 1, participants here also reported higher vividness (64.05 ± 29.66 vs. 57.33 ± 29.81; β = 5.89; 95% CI [3.41, 8.38]; *t*_74.85_ = 4.65; *p* < .001) and confidence (67.64 ± 28.10 vs. 61.68 ± 28.96; β = 4.99; [2.34, 7.64]; *t*_78.91_ = 3.68; *p* < .001) for the complete compared to the incomplete video clips (see Figure S4 in online supplemental materials).

## Experiment 3

In Experiments 1 and 2, we showed that participants make more extension errors for incomplete than complete video clips in a cued recall task. To better understand when these errors occur, we also tested memory in a recognition memory paradigm. We wanted to test whether participants would make extension errors when provided with a very strong memory cue (rewatching the video in a self-paced manner). Unlike in the cued recall task that requires active reconstruction of the studied memory trace, participants could possibly rely on their familiarity with the stimulus to respond to the recognition memory task. The procedure was very similar to Experiments 1 and 2, with the main difference being that instead of a cued recall task, participants performed a recognition memory task. Specifically, during the recognition session participants could watch and playback longer versions of the encoded video clips and they were asked to indicate the last time point they remembered from the clip. This provided us with a continuous memory accuracy error measure.

### Method

#### Participants

Sixty participants completed both the encoding and recognition session, separated by a week’s time. Four participants were excluded because they could not remember at least 30% of the video clips, leaving us with a final sample size of 56 (47 female and 11 male). Power analysis showed that with 56 participants we could achieve 80% to detect a small to medium effect size (Cohen’s *d* = 0.38: G*Power calculation for a paired *t* test). Participants’ *M*_age_ was 19.83 ± 1.89. Participants were recruited from the undergraduate psychology course at the University of Sussex and completed the experiment in exchange for course credits. The project was approved by the University of Sussex Cross School Research Ethics Committee.

#### Stimuli and Procedure

We used the same 24 video stimuli used in Experiments 1 and 2. The timings of the videos in the encoding session were identical. Here, just like in Experiment 1, we included predictability ratings in the study session. In the recognition session, we included longer cuts for each video regardless of which condition it was in. For instance, if a person had watched a 40 s video of a batter almost hitting a baseball (incomplete) then during the recognition they saw a longer version of the same video (e.g., 50 s) that showed the batter completing the action and what followed afterward. In the recognition session, participants saw longer versions of both the incomplete and complete video clips. On average videos in the recognition session were lengthened by 7.02 ± 2.22 s.

Participants always watched a longer video in the recognition session than in the encoding session. They could pause and playback the videos as much as they wished. Participants were provided with a slider that could be controlled with a mouse or keyboard arrows. The slider allowed them to navigate the timing of the video. Participants were asked to indicate when they remembered the video clip to have finished during the encoding session. They could do this by sliding to a particular time point in the video and pressing a button (see Montchal et al., 2019). Participants were asked to indicate a timing of 0 (position the slider at the beginning of the video) if they could not remember the ending of the video, even after rewatching the full, now longer, clip.

After each recognition, trial participants were asked to also provide a vividness and confidence rating. Previously participants provided these subjective ratings before completing the memory tasks.

#### Data Scoring and Analysis

The slider allowed us to have a continuous measure of memory error. For each video trial, we had the timing participants indicated at recognition and the actual timing the video ended at encoding. We subtracted from their recognition timing the actual timing at encoding, which provided us with an error measure in seconds. For instance, if one participant responded that they think the video ended at 50 s, but the video ended at 40 s, we would indicate that they were +10 s wrong. Note that the here the direction of the error is important. Positive values mean that participants recognized more of the video than was shown, and therefore made an “extension” error. On the other hand, a negative score (e.g., they indicating the video ended at 35 s when it was 40 s long; −5 difference) would indicate that they recognized less of the video then was originally shown (an “omission” error).

Apart from the raw error measure we also calculated the error as proportion of the length of the video (e.g., 35/40 and 50/40) with values above 1 indicating extension errors and values below 1 indicating omission errors.

Four participants were excluded as they recognized fewer than 40% of the video clips. Furthermore, we excluded trials where participants responded with quite large magnitude of error. We chose a cutoff ± 9 s because this was approximately 1 *SD* away from the average duration added to all the clips. We assumed that participants that only recognized frames more than 9 s away from the original ending were likely guessing on these trials. Therefore, we excluded such trials from the main analysis. In the online supplemental materials, we report analyses including guess trials.

### Results

To examine difference in our continuous measure of memory error, we fitted a linear mixed effect model including a random slope, and random intercept for participant and a random intercept for video [Memory_Error ∼ Condition + SeenBefore + (Condition | Subject) + (1 | Video)]. This model did not converge, and we dropped the random slope for condition [Memory_Error ∼ Condition + SeenBefore + (1 | Subject) + (1 | Video)]. Surprisingly, this model showed that participants showed better recognition performance for the incomplete than the complete video clips (−0.56 ± 2.60 vs. −1.50 ± 2.89; β = −0.90; 95% CI [−1.18, −0.61]; *t*_1,127.69_ = −6.16; *p* < .001). The condition difference is due to participants omitting more of the complete video clips than the incomplete (see Figure 2).

Because the main purpose of this experiment was to further investigate the tendency of participants to remember events in the incomplete condition as being extended (Experiments 1 and 2), we ran a post hoc analysis where we examined differences only in extension errors. For each trial we converted the continuous error measure into a binary measure, indicating either the presence of an extension error or the absence of an extension error. Recognition responses that exceeded a 1% of total duration of the video, such that participants indicated they remembered more than the original were treated as extension errors. All other responses were treated as nonerrors. We then examined condition differences in this new binarized dependent variable [Bin_Memory_error ∼ Condition + SeenBefore + (1 | Subject) + (1 | Video)]. This analysis did show that participants were more likely to make extension errors in the incomplete condition than the complete condition (0.31 vs. 0.22; β = −0.53; 95% CI [−0.81, −0.24]; *Z* = −3.63; *p* < .001). However, this is a post hoc analysis and it does not consider the full range of responses making it difficult to interpret if participants indeed were more likely to make extension errors during the incomplete videos. The finding that participants did not make more extension memory errors in the incomplete condition for the recognition task result contrasts with the cued-recall task in Experiments 1 and 2. This result suggests that when provided with a very strong memory cue participants can relatively accurately estimate when the video ended. Indeed, it seems to be the case that under the recognition paradigm, participants were more likely to make omission errors for the complete videos. We address this point further with Experiment 4.

Apart from memory accuracy we also examined predictability ratings, which replicated Experiment 1 showing that participants rated the incomplete video clips as having a more predictable continuation than the complete video clips (68.22 ± 26.98 vs. 51.70 ± 31.27; β = −16.81; 95% CI [−19.74, −13.89]; *t*_1,132.89_ = −11.28; *p* < .001). We observed higher vividness for the complete compared to the incomplete videos (66.95 ± 25.45 vs. 63.54 ± 27.36; β = 2.99; 95% CI [0.23, 5.74]; *t*_49.71_ = 2.13; *p* = .038). Unlike the previous experiments, we did not observe any differences in confidence between the incomplete and complete videos (60.79 ± 29.16 vs. 61.55 ± 26.60; β = 0.67; 95% CI [−2.17, 3.52]; *t*_50.95_ = 0.46 *p* = .644). We note that vividness and confidence ratings were provided after the recognition trial, which might have affected the results (see Figure S5 in the online supplemental materials).

## Experiment 4

In the previous cued recall experiments, we observed that participants make more extension errors for the incomplete videos compared to the complete clips. We note that the length of video clips was not matched across conditions in the previous experiments, which could be a confounding factor. Here we used novel video clips which we matched in length across the conditions. The aims of Experiment 4 were to examine, (a) whether this effect would generalize to other video clips, and (b) whether participants would falsely recall additional details about the ending of the video, if the video itself ended immediately after the beginning of a new scene (following a salient event boundary).

To address these questions, we used stop-motion video clips showing different actors performing everyday activities (e.g., unlocking a bike and setting the table). Some of the clips were available online and were used in a different project (Ben-Yakov et al., 2021) and some were shot by one of the authors (Dominika Varga). We again included video clips that were interrupted before an action was completed (incomplete condition). Critically, instead of a complete condition we included an updated condition in this experiment (see Figure 1). The updated clips were edited so that they showed the completed action and then an additional new scene including the same actor performing another action. For example, in one of the clips a man is unlocking his bike, putting a seat on his bike, and putting a helmet. In the incomplete condition, the clip is cut just as the actor is about to put the helmet on his head. The updated version shows the actor put on his helmet, which is followed by a scene change where the actor is shown sitting on a bench wiping sweat from his forehead (see https://tinyurl.com/a5epxzab). This new scene is shown only for a few seconds. We preregistered our hypotheses that participants will make more extension errors for the incomplete condition compared to the updated (https://osf.io/fuyva). We further hypothesized that participants would make more omission errors (fail to recall the last action–putting a helmet or wiping his forehead) for the updated videos compared to the incomplete videos.

### Method

#### Participants

We recruited 105 (68 female, 36 male, and one nonbinary) participants from Prolific (https://www.prolific.co/), who completed both sessions of the experiment online. The *M*_age_ was 28.05 (± 6.95). We note that due to an oversight the age range was not set to our preregistered age range of 18–35 years, when recruiting participants from Prolific. As a result, 12 participants out of the 105 (11%) had an age above 35 (40.91 ± 4.67; range = 36–51). These participants were included in the data scoring and further analyses procedures. We excluded 22 participants because they remembered fewer than 40% (10) of the videos. We excluded one additional participant as they did not follow the instructions. In the final analyses, there were 84 participants. Power analyses based on effect sizes in Experiments 1 and 2 suggest we can achieve 99% power to observe a medium-sized effect (Cohen’s *d* = 0.42; Sánchez-Meca et al., 2003). We did not have an effect size estimate for the omission errors, however, preregistered simR simulations suggested that with 80 participants we could observe an effect 5.5 times smaller (Cohen’s *d* = 0.22) than the previously observed effect for memory extension errors with 71% power (https://osf.io/fuyva). Participants were compensated with £9 for completing both sessions. The research project was approved by the University of Sussex Cross School Research Ethics Committee.

#### Stimuli

We used 24 videos of actors performing everyday activities. Spatial location varied between the videos and showed actors performing activities inside (e.g., baking) and outside (e.g., washing car). Six of the video clips were recut from videos used by Ben-Yakov et al. (2021) the remaining 18 were shot by one of the authors (Dominika Varga). The video clips were stop-motion presenting six frames per second and had a mean length of 29.87 (±8.74; range = 18.14–53.6).

For each video clip, we created two versions that manipulated the information presented in them. Unlike Experiments 1–3, here we maintained the length of the video clips under different conditions. This was done by using a sliding window approach, such that videos in the incomplete condition started earlier than videos in the updated condition. The videos were cut to end before or shortly after an action was completed. We selected videos that had an additional scene change after the last action in the first scene was completed and would show the start of a new event. On average the duration of the new event after the scene change (e.g., wiping the forehead) was 1.99 (±0.57) s. On average, the incomplete video clips were shifted 4.96 (±1.08) s forward in time to include the completed action, the scene change, and the short event after the boundary. We varied which videos are in the incomplete condition and which were in the updated condition, by creating two counterbalancing lists. In each list, 12 videos were in the incomplete condition and 12 were in the updated condition.

We used a title for each clip and a still image of the first frame of the clip, blurred with a two-dimensional Gaussian kernel with *SD* of 25, as a retrieval cue in the recall session. The blurring of the first frame was done in order to provide participants with a partial cue and prevent them from simply describing stereotypical actions associated with the spatial location present in the first frame.

#### Procedure

The encoding procedure was identical to Experiments 1 and 2, apart from using different 24 video clips. We did not include a predictability rating after each clip. An additional difference was that we included an example video in the instructions and showed an ideal memory response for the clip, to encourage participants to recall as much as they could.

One week after completing the encoding phase, participants were asked to complete the recall phase. During the instructions, participants again saw the example video clip and example memory response and were asked to try to remember as many details and actions from the clips as possible. During the recall phase, participants had to remember the clips in a random order. Participants’ memory for a specific video was cued with the title and the blurred first frame from the video. After the memory cue, participants were asked to provide vividness and confidence ratings from 0 to 100 for each video. After providing the subjective memory ratings, participants again saw the title and the blurred first frame and were asked to type in their memory for the video in as much detail as possible. Participants were also instructed to focus on how the video clip ended. After entering their response, they saw a fixation cross for a randomly jittered duration between 700 and 2,500 ms, which was followed by a pause trial. The recall phase was a self-paced task.

#### Data Scoring and Analysis

We scored responses using similar strategy to Experiments 1 and 2. Responses were rated by author Dominika Varga. A subset of the ratings was additionally rated by author Petar P. Raykov to ensure consistency across experiments. Initially, for each trial we scored responses as Remember/Forgotten if the participant was able to remember something specific about the video clip that was not simply referring a generic situation represented by the title (e.g., making tea). We also scored extension memory errors as present or absent (1 or 0), depending on whether participants recalled something about the end of the video that did not occur. Unlike previous experiments, we also scored omission errors from the end of the video clips as either present or absent (1 or 0). An omission error was scored if participants did not mention the last action or scene in the video. Both extension errors and omission errors were only scored on trials that were remembered by participants.

We initially preregistered that we would do both a multinomial mixed-effect logistic regression, and two separate mixed-effect logistic models treating each memory error separately. In contrast to our preregistration, we did not run a full multinomial mixed effect logistic regression, because this did not treat the memory error types as independent and affected our reference category. For instance, in this new combined memory error measure, the lack of omission error would count only if participants also did not make an extension error and vice versa. Therefore, as preregistered we opted for running two separate mixed effect logistic regressions for each memory error type [Extension ∼ Condition + (Condition | Subject) + (Condition | Video)] and [Omission ∼ Condition + (Condition | Subject) + (1 | Video)].

### Results

Participants remembered 72.9% of trials in the cued memory task. Fitting a mixed effect logistic model [Extension ∼ Condition + (Condition | Subject) + (Condition | Video)]—we found that participant made more extension memory errors in the incomplete condition compared to the updated condition (26.1% vs. 7.3%; β = −1.64; 95% CI [−1.98, −1.29]; *Z* = −9.37; *p* < .001; see Figure 2). These results replicate our previous experiments and demonstrate that the higher proportion of extension errors generalized to a different set of videos that had matched duration.

**Figure 3 fig3:**
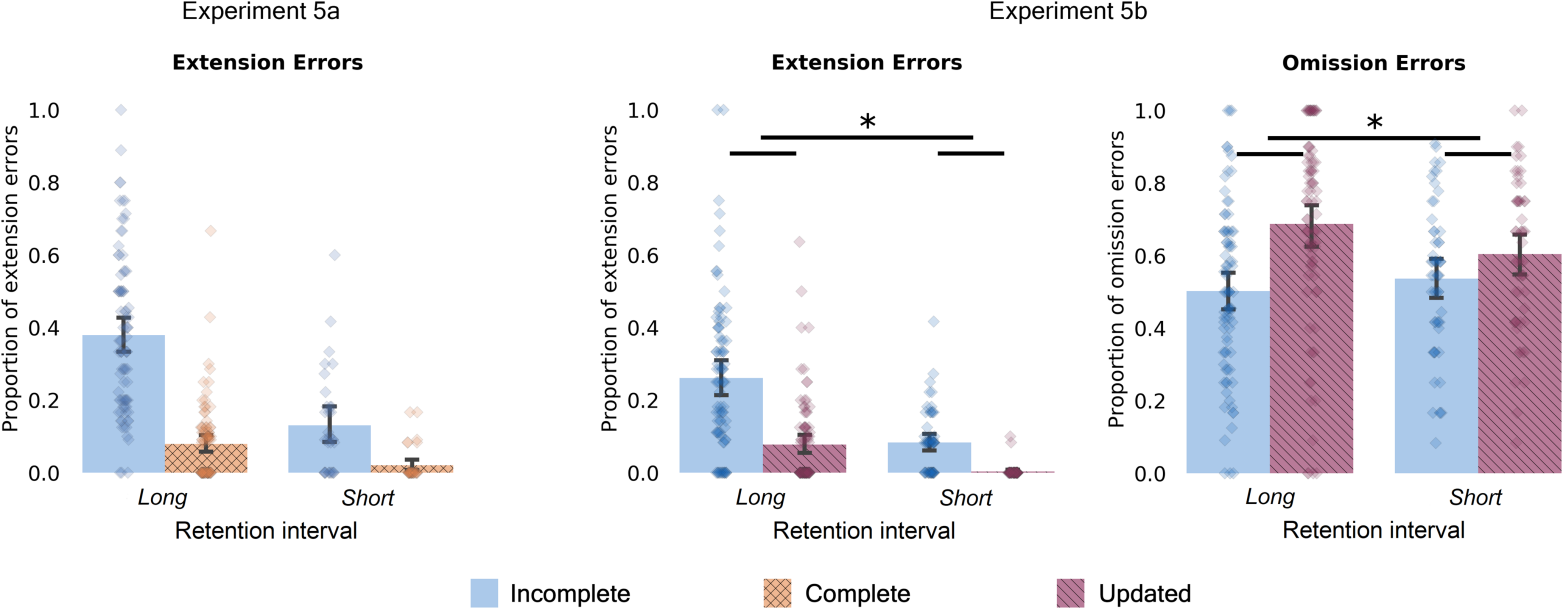
Effect of Delay *Note.* The figure shows extension and omission errors when participants were tested in the same session as an encoding (short retention interval; Experiments 5a and 5b) compared to the previously reported results that tested memory after one week (long retention interval; Experiments 2 and 4). Experiment 5a: Participants made more extension errors for the incomplete compared to the complete videos when they were tested with cued recall immediately after the study session. Additionally, we found a main effect of delay, such that participants made more errors at the long compared to the short retention interval. There was no significant interaction between delay and condition. Experiment 5b: At the short retention interval, participants made more extension errors for the incomplete compared to the updated videos. We additionally observed a main effect of delay with participants making more errors at the long retention interval. There was a significant interaction between delay and condition, but that should be interpreted with caution as there was a floor effect in the updated condition of the short retention interval. Participants made more omission errors for the updated videos compared to the incomplete videos. There was additionally a significant delay by condition interaction, showing that omitting the new scenes for the updated condition was more pronounced at the longer retention interval. Error bars represent 95% confidence intervals of the mean. See the online article for the color version of this figure. * *p* < .05. ** *p* < .01. *** *p* < .001.

In this experiment, we also included a measure of how likely participants were to make omission errors about the ending of the clip. We fitted a mixed effect logistic model examining likelihood of making omission errors [Omission ∼ Condition + (Condition | Subject) + (1 | Video)]. As in our preregistered hypothesis, we found that the participants were more likely to make omission errors for the updated condition when compared to incomplete condition (66%—updated vs. 48.8%—incomplete; β = 0.95; 95% CI [0.60, 1.28]; *Z* = 5.45; *p* < .001; see Figure 3 under Experiment 5b delayed condition). These results suggest participants may have a tendency to remember coherent events, by either falsely adding information to incomplete events or by omitting information from just started events.[Fig fig3]

Unlike Experiments 1 and 2 here, we did not observe differences in Remembered/Forgotten responses across conditions (73.7% incomplete vs. 72.1% updated; β = −0.01; 95% CI [−0.29, 0.27]; *Z* = −0.049; *p* = .96). We used different video clips and a different cueing. We also did not observe any differences in subjective measure of vividness between incomplete versus updated (38.01 vs. 37.88; β = −0.16; 95% CI [−2.16, 1.83]; *t*_1,389.00_ = −0.161; *p* = .87) and memory confidence (37.98 vs. 36.80; β = −0.83; [−2.82, 1.14]; *t*_1,379.93_ = −0.83; *p* = .41). This raises the possibility that the objective and subjective memory differences in Experiments 1 and 2 were due to the videos in the complete condition being slightly longer.

In the online supplemental materials, we describe a separate experiment using the same clips but including only an encoding session. Critically, we ran this experiment to examine whether participants could more confidently predict what would happen next in the incomplete videos compared to the updated videos. We found that predictability ratings were higher for the incomplete compared to the updated clips (see Figure S6 in the online supplemental materials), in line with previous findings (Eisenberg et al., 2018; Zacks et al., 2011).

## Experiment 5

In previous experiments, we showed that after a week, participants are more likely to make extension errors for video clips interrupted before an expected ending, but more likely to make omission errors when video clips are interrupted shortly after an expected ending. If such errors disproportionately increase at long compared to short delays (indicating a delay by condition interaction), it would suggest that these effects were mainly driven by reconstructive processes during memory retrieval. However, if the relationship between errors and conditions remains constant across both long and short delays (i.e., in the absence of a delay by condition interaction), it cannot be firmly concluded that retrieval processes are solely responsible for driving the errors. Instead, it would suggest that both retrieval and encoding processes might be involved. We, therefore, use the same videos and conditions as in Experiments 2 and 4, but instead memory was tested in the same session immediately after the video encoding phase. There was still a short delay between watching and retrieving the clips, but much shorter than the week delay used in the previous experiments. We then ran analyses to compare the results across studies to examine how delay affected memory errors. Specifically, we compared the results from Experiment 2 to Experiment 5a, and the results of Experiment 4 to the results of Experiment 5b.

### Method

#### Participants

Forty-one participants and 60 participants recruited from Prolific completed Experiments 5a and 5b, respectively. Using the same criteria as above we excluded participants that remembered fewer than 40% of the clips. This resulted in excluding three participants from Experiment 5a and four from Experiment 5b. We had 38 (19 female and 19 male) participants for Experiment 5a with *M*_age_ 26.57 (±5.15), and 56 participants aged 29.65 (±5.28). Note for Experiment 5b due to a coding error, we had the demographics data for 38 people (15 male and 23 female). Power analyses using simR showed that with 40 people we would have about 89% to detect a medium effect size of Cohen’s *d* = 0.5 (logOdds = 0.94), based on the previously observed omission errors. The experiments were preregistered before any data collection was initiated (https://osf.io/ybp52). The research project was approved by the University of Sussex Cross School Research Ethics Committee.

#### Stimuli and Procedure

In Experiment 5a, participants were shown stimuli used in Experiment 2 during the encoding. The procedure was nearly identical to Experiment 2 with the difference that participants recalled video clips immediately after encoding all 24 of them. In Experiment 5b, a separate group of participants was shown stimuli used in Experiment 4 during the encoding session, and the procedure was nearly identical to Experiment 4 with the difference that participants recalled video clips immediately after encoding all of them. Memory for the clips was tested in random order. The task was a self-paced cued recall task.

#### Data Scoring and Data Analysis

Data scoring procedure for Experiment 5a was identical to Experiment 2, and the data scoring procedure for Experiment 5b was identical to Experiment 4 described above. Petar P. Raykov rated responses for Experiment 5a and Dominika Varga rated responses for Experiment 5b. For participants in Experiment 5a, we scored extension errors about the end of video clips in the incomplete and complete conditions, and for participants in Experiment 5b, we scored extension and omission errors about the end of video clips in the incomplete and updated conditions. We fitted logistic mixed effect models using lme4 (Bates et al., 2014) package available in R. We excluded participants that recalled fewer than 40% (10 of the videos). The logistic mixed effect regression models in Experiment 5a had the same parameters as in Experiment 2; in Experiment 5b they had the same parameters as in Experiment 4.

In addition to examining the difference in errors between conditions at the short retention interval, we also examined whether there were any differences in extension and omission errors when comparing short to long retention interval—for example, [Extension ∼ Condition × Delay + (Condition | Subject) + (1 | Video)].

### Results

Similarly, to our previous results from Experiment 2, but with a much shorter retention interval, we found that participants in Experiment 5a made more extension memory errors in the incomplete condition compared to the complete condition at the short delay (12.4% vs. 2.1%; β = −2.45; 95% CI [−4.15, −0.76]; *Z* = −2.84; *p* = .004). We found a significant main effect of delay (short vs. long), such that overall participants made more extension errors in the long retention interval compared to the short (β = 1.60; 95% CI [1.13, 2.06]; *Z* = 6.70; *p* < .001; see Figure 3). However, we did not find a significant interaction between delay and condition, such that the difference between extension errors across incomplete and complete conditions was similar for both retention intervals (β = −0.25; 95% CI [−1.06, 0.56]; *Z* = −0.614; *p* = .53).

In Experiment 5b, we found that participants made more extension errors in the incomplete condition compared to the updated condition at the short retention interval (8.4% vs. 0.3%; β = −3.51; 95% CI [−3.55, −2.26]; *Z* = −4.81; *p* < .001; Extension ∼ Condition + (1 | Subject) + (1 | Video)). When comparing results across retention intervals we observed a main effect of delay, such that participants overall made more extension errors when recalling 1-week later rather than immediately after encoding all the clips (β = 1.57; 95% [1.08, 2.06]; *Z* = 6.3; *p* < .001). There was a significant interaction between condition and delay (β = 1.69; 95% CI [0.22, 3.16]; *Z* = 2.26; *p* = .023). However, because of floor effects during the updated condition at the short retention interval, this interaction should be interpreted with caution. We note that at the short delay, the incomplete versus updated extension error difference was (Δ8.1%). In the 1-week delay experiment the incomplete versus updated extension error difference was (Δ18.8%).

When focusing on the omission errors in Experiment 5b, we found that participants made significantly more omission errors in the updated compared to the incomplete condition (59.5% vs. 52.5%; β = 0.32; 95% CI [0.07, 0.57]; *Z* = 2.48; *p* = .013); [Omission ∼ Condition + (Condition | Subject) + (1 | Video)]. Additionally, when comparing omission errors across retention intervals we found a significant interaction between delay and condition such that the difference in omission errors (incomplete vs. updated) was smaller at the short retention interval when compared to the long (β = 0.53; 95% CI [0.12, 0.95]; *Z* = 2.52; *p* = .012). We did not find a significant main effect of delay (β = −0.12; 95% CI [−0.44, 0.21]; *Z* = −0.687; *p* = .492).

Overall, these findings are in line with prior work showing that memory errors increase with increased retention intervals. Nonetheless, they show that errors can occur even at very short memory delays, suggesting that encoding-based processes may contribute to making extension errors.

## Results Summary

Across multiple experiments, we observed that participants make more extension errors when the encoded videos were interrupted abruptly. In Experiments 1 and 2, we found that participants had better memory for the complete compared to the incomplete videos. This is in line with some research showing worse memory when events are interrupted at unnatural boundaries Boltz (1992; Flores et al., 2017; Gold et al., 2017; Schwan & Garsoffky, 2004). However, we note we did not observe this effect in Experiment 4 where the duration of the videos was matched across conditions so further research is needed to understand the generalizability of the effect. In the online supplemental materials, we show results from post hoc analyses that examined the relationships between subjective measures (predictability, vividness, and confidence) and memory errors. We found evidence suggesting that predictability ratings were not associated with the probability of making an extension error. Furthermore, we did not find a clear-cut association between subjective memory measures and extension errors. Some analyses showed that lower subjective memory measures were associated with more memory errors.

## Discussion

In a series of experiments, participants were much more likely to erroneously recall the endings of events that ended at unexpected time points, compared with events that stopped at a coherent event boundary. When the video clips were interrupted just before an expected endpoint, participants often falsely recalled additional details going beyond the last interrupted action. Conversely, when video clips were interrupted shortly after an event boundary, at the very beginning of a new scene, participants often omitted the entirety of the new scene from their recall. These effects were still present, albeit to a lesser extent, when participants were tested within the same session, compared to after 1 week. However, we did not see the same tendency to extend videos in the incomplete condition when memory was tested using a recognition paradigm. Taken together our results show that interrupting events at time points, which are inconsistent with typical event structure leads to memory distortions in recall. Our interpretation of these results is that prior knowledge about the typical structure of events biases recall of the video clips toward completed events.

One of our key findings is that interrupting events just before their typical ending increases the likelihood of recalling false details beyond the actual endpoint of the video clips (see Figure 2). We will refer to this as “event extension,” because it shares similarities with the well-established phenomenon “boundary extension,” where people tend to recall the background of a closely cropped photograph of an object as being more extensive than it actually was (Intraub, 2012; Intraub & Richardson, 1989). More generally, there are many examples of humans extrapolating beyond what was physically presented to represent its likely future state (Hubbard, 2015). For example, “representational momentum” is characterized by remembering the location of a moving target as being further along its trajectory than when it was last observed (Freyd & Finke, 1984; Thornton & Hubbard, 2002). Our event extension finding shows that people have a tendency to extend the bounds of complex unfolding events by recalling inferred details about their endings.

One explanation for event extension is that participants’ strong expectations about how the interrupted actions were going to unfold might be incorporated into their memory for the event. Studies have shown that people can infer the goal of an action and use this information to predict the action’s target; for example, people produce anticipatory eye movements toward the target object before it is acted upon (Eisenberg et al., 2018; Flanagan & Johansson, 2003; Kamide et al., 2003). In our experiments, people could have similarly used inferred goal information to anticipate the outcome of interrupted actions, as well as more general schematic knowledge about how similar situations typically end. In our Experiments 1 and 3, predictability ratings support the proposal that participants were readily able to anticipate what was likely to happen next in the interrupted clips.

If participants incorporate their expectations about the endings of events into their memory, it is important to address whether this happens during encoding or when retrieving the events. Most of our experiments tested memory after one week, but in Experiment 5, we tested people within the same session in which they encoded the videos. We reasoned that if extension and omission errors were disproportionately greater after one week (quantified by a condition by delay interaction), then this would be evidence that memory distortions were predominantly associated with memory retrieval processes. We did find that memory errors were increased in both the incomplete and updated conditions compared to the complete condition, and that memory errors increased with the retention interval. These findings are broadly in line with evidence that memory intrusions tend to increase with longer delays between the study and retrieval phase (Bartlett, 1932; Bergman & Roediger, 1999; Brewer & Treyens, 1981; Kleider et al., 2008; Rubínová et al., 2021; Wagoner & Gillespie, 2014; Wheeler & Roediger, 1992). However, there was no significant interaction between delay and extension errors in the incomplete versus complete conditions, and therefore we conclude that memory biases may be introduced by processes at encoding and retrieval.

It is well established that schemas play a role in organizing and structuring how incoming information is encoded into memory (see Alba & Hasher, 1983). Indeed “event schemata are proposed to influence event segmentation (Franklin et al., 2020; Zacks, 2020; Zacks et al., 2007), and it has been shown that neural correlates of event scripts (Baldassano et al., 2017; Masís-Obando et al., 2022) and of schematic knowledge about people (Raykov et al., 2020, 2021) are active when encoding new events. Furthermore, research into boundary extension, representational momentum, and related phenomena has revealed that online representations of external stimuli can incorporate extrapolated information that is not physically present (Bar, 2004; Hubbard, 2015; Hubbard et al., 2010; Hymel et al., 2016; Intraub, 2012; Intraub & Richardson, 1989; Papenmeier et al., 2019; Strickland & Keil, 2011). Nevertheless, anticipating the trajectory of a moving dot is very different from representing how people and/or objects might interact in complex lifelike situations.

How could incorrect versions of the endings of the videos become encoded and later retrieved? In our experiments, in the incomplete condition, it is likely that when the video abruptly stops, people infer what might have happened next and encode this imagined ending. Therefore, the recall errors we observed could reflect a failure of “reality monitoring” (Johnson & Raye, 1981). When recalling the events the memory for the imagined ending would compete with the memory for the true ending and may be incorrectly selected. Memories for episodic details are forgotten relatively quickly (Conway et al., 2001), and therefore, after a week it would be harder to distinguish the true ending from a plausible competing memory trace, resulting in increased numbers of extension errors compared with errors made at a short retention interval. A similar mechanism might explain omission errors in the updated condition. Here, the videos show an event boundary at the end of the first scene, before a related second scene is briefly shown. Participants are likely to encode the first scene to long-term memory at the event boundary (Ben-Yakov et al., 2013), before also encoding the second scene after that finishes. Therefore, at retrieval, there are competing memory traces to select from: One which only includes the first scene of the video and another which correctly includes both scenes. However, if this reality monitoring explanation is correct, why should participants show such a strong bias to select the incorrect memory for the video? We suggest that this is due to people’s expectation that events have a predictable structure, with clearly defined endings. This will bias people to recall events as if they ended at an event boundary and retrieve a representation of the event that satisfies this expectation.

An alternative explanation for the memory errors that we found is that they are partially due to retrieval mechanisms. Under the assumption that memory retrieval is reconstructive (Bartlett, 1932; Schacter, 2012) and reflects a combination of event-specific details, as well as more schematic gist-like information (Bromis et al., 2021; Spens & Burgess, 2023), then plausible endings for the videos in the incomplete condition might be generated on-the-fly during the recall tests. While the specific content of the falsely recalled endings would be based on familiarity with the particular situations depicted in each video, the over-arching driver of the memory errors would be the expectation that events typically have coherent endpoints. If new endpoints to the videos are created during memory recall, then this would imply that novel event units and their associated boundaries are created at retrieval—something which has been suggested but for which there is little empirical support (Zacks, 2020). Since we found that increased extension and omission errors were associated with the incomplete and updated conditions respectively, at both short and long retention intervals, it is likely that processes at encoding and retrieval played a part. Overall, it is therefore not possible to support the reality monitoring explanation over the memory reconstruction explanation on the basis of our results and both may play a significant role in explaining false recall in our experiments.

Our results speak to the role of prediction errors in shaping memory. Recently, Sinclair and colleagues showed that interrupting expected action-outcome contingencies in video clips was associated with increased false memories (Sinclair & Barense, 2018; Sinclair et al., 2021). A key difference from their studies and ours was that expectancies about the videos were established by having participants watch all videos first. The authors found that reexperiencing events in an interrupted way made memories more vulnerable to intrusions from schematically similar events. Our findings show that experiencing events in an interrupted way in the first place can distort memories. Moreover, interrupting events can lead to false memories generated from inherent biases to extend or omit details to create coherent endings to the events, not just to intrusions from similar episodes. Overall, our findings are consistent with the idea that interrupting expected outcomes causes memory distortions.

There is also evidence that expectation violation leads to improved memory (Antony et al., 2021; Ben-Yakov et al., 2021; Chen et al., 2015; Greve et al., 2017; Hermann et al., 2021; Stawarczyk et al., 2020; Wahlheim et al., 2022). Most prior work tested the effect of prediction errors on learning by introducing new information that is incongruent with prior predictions (e.g., video clip showing brushing teeth with a rhubarb rather than a toothbrush) and showed that often people remember incongruent occurrences better than expected information. An important distinction between our design and prior work is that here we induced prediction errors simply by omitting information about what would happen next. Exactly how our predictions are violated, by omission or commission of information, is likely to have a different effect on learning (Gershman et al., 2012; Kim et al., 2014, 2017; T. A. Smith et al., 2013).

Finally, it is important to note that extension errors were only noted when memory was tested using a recall, not a recognition, paradigm. In Experiment 3, participants rewatched the clips and indicated where they remembered the endings to be. In this experiment, participants tended to place the ending earlier than when it occurred, and this underestimation of the endpoints was most marked for videos in the complete condition. These findings are broadly consistent with research showing that forward inferential errors are rarely made in recognition memory studies; people do not falsely recognize unseen consequences of actions as having been seen (Hannigan & Tippens Reinitz, 2001; Papenmeier et al., 2019). The finding of a greater underestimation of the endpoints in the complete condition is surprising and difficult to interpret, although we note that the difference between the conditions was small (the underestimation was 1.5 and 0.6 s for the complete and incomplete conditions respectively). Overall, the results of Experiment 3 suggest that the errors seen on our recall tests are likely to be due to participants self-generating plausible endings to the videos (either during encoding or retrieval). However, when viewing the “real” continuations of the videos, participants rarely falsely recognized additional details.

### Constraints on Generality

We ran multiple experiments replicating a novel false memory effect using video clips. Participants were from a population of U.K. undergraduate psychology students and a population of participants sourced from Prolific, where the only constraints were that participants were native English speakers and did not have language impairments. We replicated the effect using two sets of videos, one sourced from the internet and one set created by the authors. We note that here we tested only young adults (up to 35 years) so it is possible that the effect will be attenuated or actually possibly increased in older adults. A further limitation is participants were tested online without direct experimental supervision.

## Conclusion

In a series of experiments, we found a bias to recall coherent, completed events, even if this resulted in memories that were inaccurate records of what had been presented. Our paradigm involved interrupting video clips of everyday activities before or after natural endpoints in the actions of the protagonists. When the clips were interrupted just before a goal-directed action was completed, people often falsely recalled additional details about the expected outcome of the scene, which filled the gap between the actual endpoint and its natural conclusion. We refer to this as event extension. On the other hand, interrupting a clip just after an event boundary biased participants to omit details of the new scene that followed the boundary. Overall, we suggest that memory retrieval is biased to retrieve completed events which correspond to our expectations about typical event structure (where events have beginnings, middles, and ends).

## Supplementary Material

10.1037/xge0001462.supp
