# Supplementary material for: False Memories for Ending of Events
Source: J Exp Psychol Gen. 2023 Aug 31;152(12):3459–75. doi: 10.1037/xge0001462 (PMC10694998; doi:10.1037/xge0001462)
Supplement: Supplementary file 1 [file xge0001462.pdf]

Supplementary Materials

Experiment 1

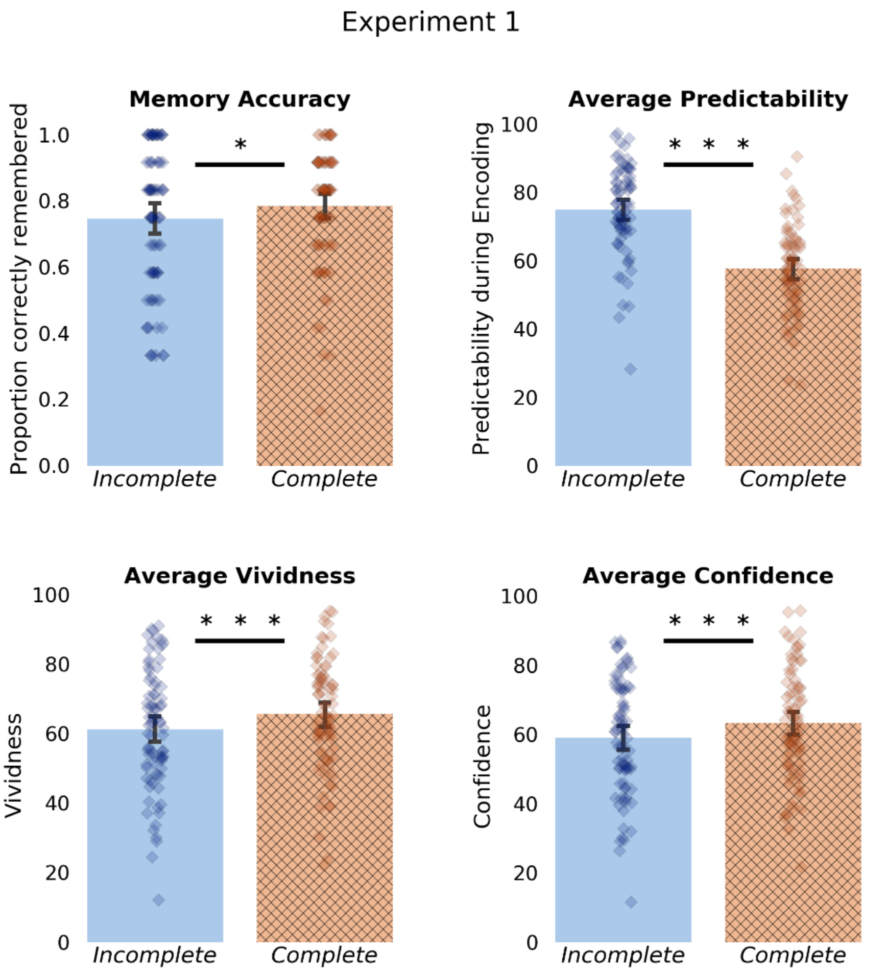

Supplementary Figure 1 **Experiment 1:** Top Panel: (Left) Participants remembered more videos in the *Complete* condition compared to the *Incomplete* condition. (Right) During the encoding phase, participants rated that they could more easily predict how the *Incomplete* videos will continue compared to the *Complete* videos. Bottom Panel: participants rated their memory as more vivid (Left) and more confident (Right) for videos in the *Complete* condition compared to the *Incomplete* condition. Error bars represent 95% confidence intervals of the mean. *p<0.05; ***p<0.001.

In experiment 1, during the encoding session, we collected measures on predictability of the videos. Specifically, we asked participants to rate how confidently they felt they could predict what would happen next in the video (after it finished). In a post-hoc analysis we examined whether our predictability measure predicted probability of making an extension error after accounting for the condition difference between the *Incomplete* and *Complete* videos. Therefore, we fitted a mixed effects logistic regression model as (Extension ~ Condition + SeenBefore + Predictability*Condition + (Condition| Subject) + (1|Video)). We used brms package to estimate the model parameters using Bayesian inference, which also allowed us

to use Bayes Factors to compare between models with and without including Predictability.

For both models we used a Bernoulli distribution with a logit link function resulting in standard logistic mixed effect model. Models were fitted with uninformative (flat) priors on the fixed slope effects and default student-t distribution priors for the variance components as implemented in brms package. We ran four chains each with 10000 iterations to estimate the models using 1000 warm-up samples and examined chain convergence. Using the bayes_factor function in brms we compared the model including predictability and condition interaction to a model that did not include predictability as a regressor. We found evidence in support of the null model that $BF_{01} = 9.42$, suggesting that our predictability measure does not explain variance in the extension errors after accounting for the condition factor.

Table S1 Population Level Effects from model excluding predictability

	Estimate	Est.Error	l-95% CI	u-95% CI	R-hat
Intercept	-0.37	0.18	-0.73	-0.01	1
Condition	-2.44	0.23	-2.92	-2.03	1
SeenBefore	-0.10	0.35	-0.79	0.57	1

Table S2 Population Level Effects from model including predictability (main effect and interaction).

	Estimate	Est.Error	l-95% CI	u-95% CI	R-hat
Intercept	-0.36	0.19	-0.73	0.00	1
Condition	-2.45	0.24	-2.96	-2.02	1
SeenBefore	-0.10	0.35	-0.80	0.58	1
Predictability	-0.01	0.12	-0.24	0.22	1
Condition:Predictability	0.02	0.18	-0.34	0.38	1

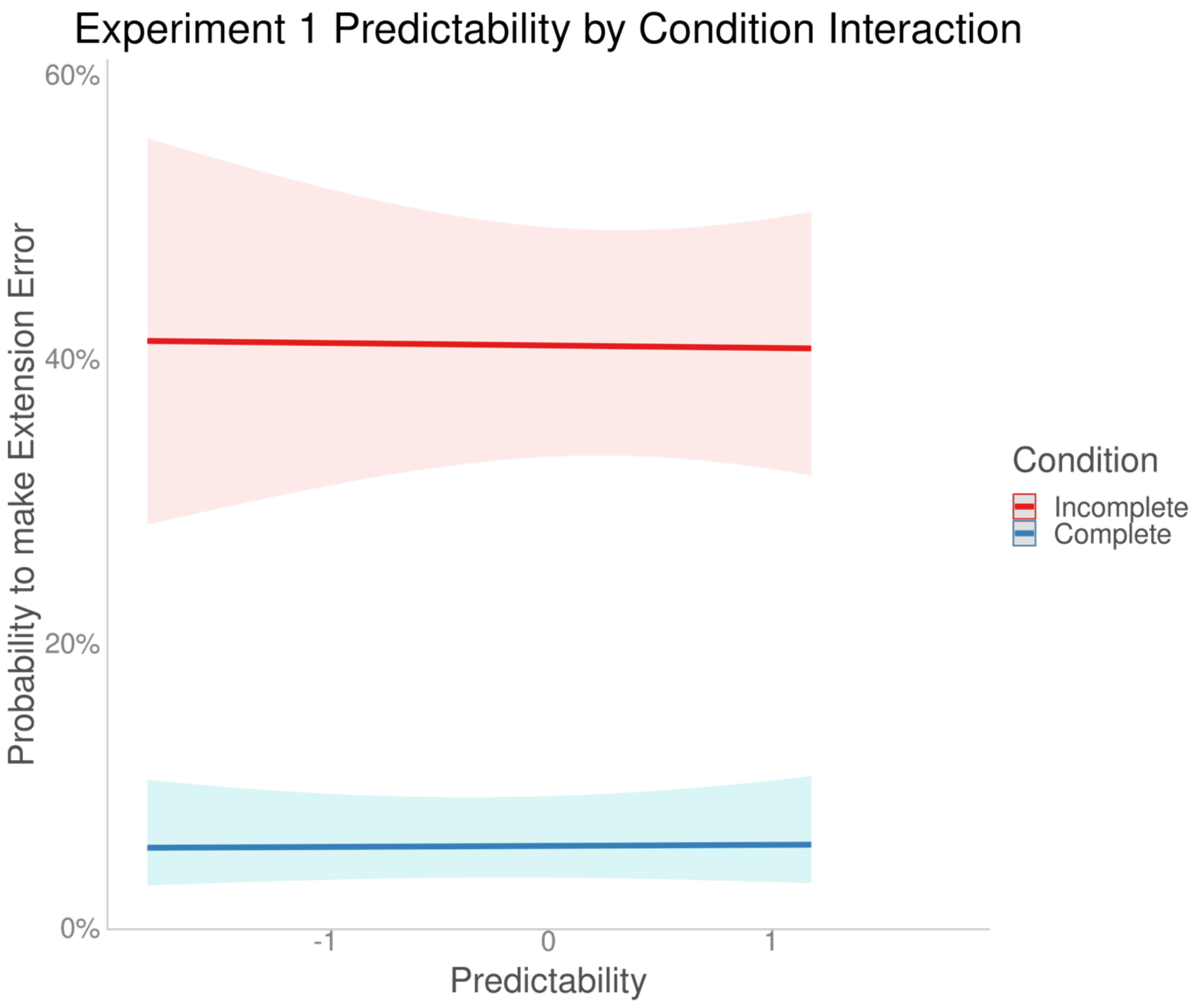

Supplementary Figure 2. **Experiment 1:** Predicted extension errors for different predictability and condition levels. Plot shows predicted values of extension errors of fitted model (Extension ~ Condition + Seen_Before + Predictability*Condition + (Condition | Subject) + (1|VideoID)). Line represents average effects and shaded area represents confidence intervals.

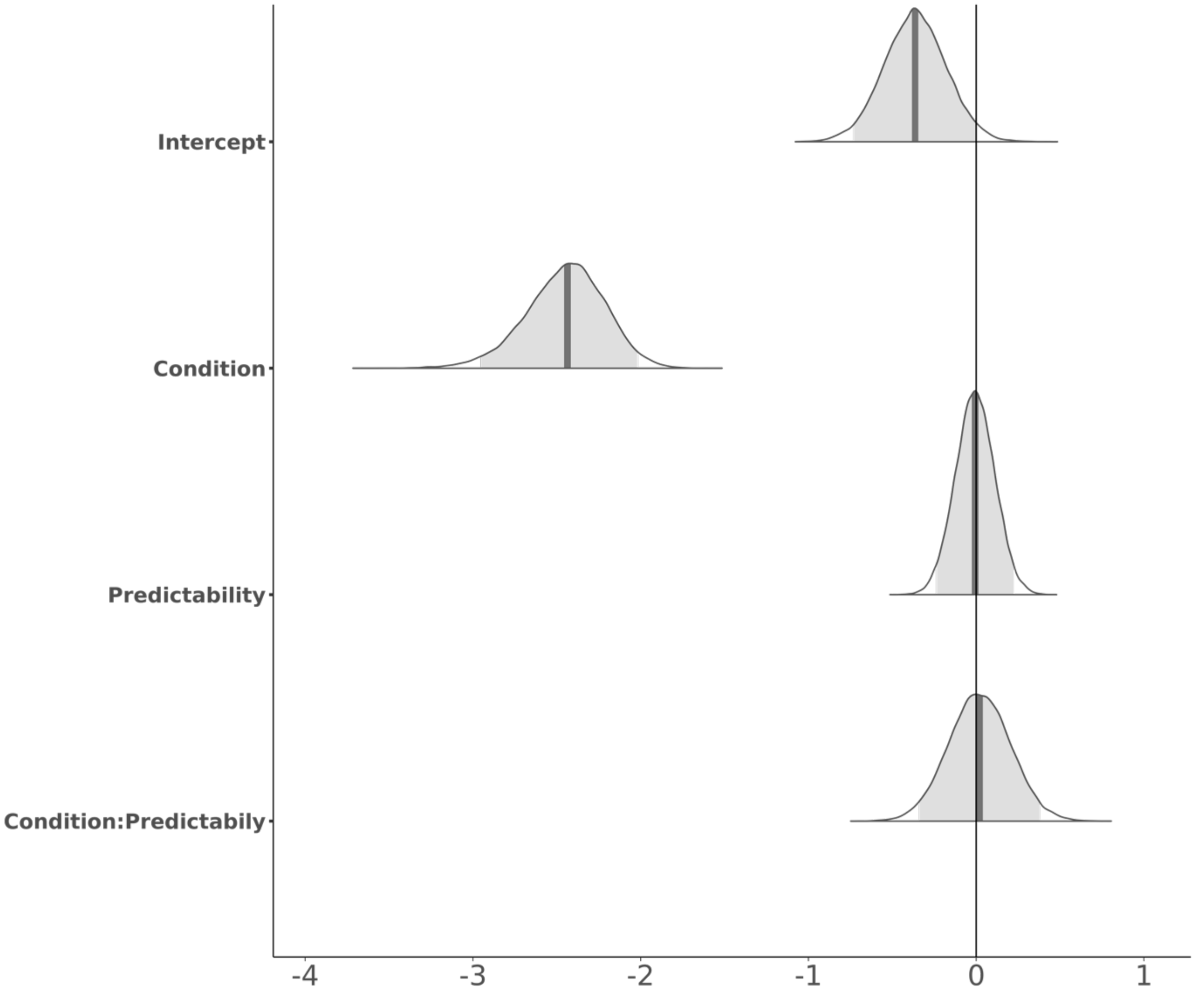

Supplementary Figure 3. **Experiment 1***:* Estimated Posterior distributions for the condition and predictability effects. Dark shaded area shows median of the posterior distribution, the grey shaded area shows the 95% Highest Density Interval. Distributions centered at 0 suggest no effect of the predictor on extension memory errors. Note x-axis is shows logOdds.

Descriptive relationships between variables

For completeness, we report association between vividness, confidence, and predictability in the separate experiments. Note these are post-hoc analyses, unrelated to our main hypotheses. Where we report correlations, they were performed by taking into consideration the multilevel structure of the data. We used the '*correlation*' package recently developed by Makowski and colleagues (2020).

Experiment 1

First, we examined the pairwise correlations between vividness, confidence and predictability. This was done for all trials. We found that vividness and confidence were highly correlated. Furthermore, vividness and confidence were also correlated to predictability to a smaller extend.

Table S3 Pairwise correlation between vividness, confidence and predictability in Exp. 1 including all trials

Variable 1	Variable 2	r	CI	$t\text{-stat}_{(1798)}$	p-value
Vividness	Confidence	0.89	[0.88, 0.90]	82.50	<0.001
Vividness	Predictability	0.14	[0.09, 0.18]	5.81	<0.001
Confidence	Predictability	0.13	[0.09, 0.18]	5.60	<0.001

Focusing on the remembered trials that entered the final model, we observed high correlation between vividness and confidence. Predictability was not strongly correlated with either vividness or confidence. Although we note that the correlations were significant.

Table S4 Pairwise correlation between vividness, confidence and predictability in Exp. 1 including all trials

Variable 1	Variable 2	r	CI	$t\text{-stat}_{(1377)}$	p-value
Vividness	Confidence	0.84	[0.82, 0.85]	57.19	<0.001
Vividness	Predictability	0.07	[0.02, 0.13]	2.71	0.007
Confidence	Predictability	0.08	[0.03, 0.13]	2.94	0.007

Given the high correlation between vividness and confidence we computed a combined measure. This was done simply by averaging for each trial the vividness and confidence. This was referred to as Subjective_Memory score. We then computed a logistic mixed effect model predicting extension errors from the condition, Subjective_Memory score and their interaction (Extension ~ Condition + SeenBefore + Subjective_Memory + Condition*Subjective_Memory + (Condition | Subject) + (1 | Video)).

We found that higher Subjective_Memory ratings were associated with less extension errors (ß = -0.289; CI [-0.53 -0.05]; Z = -2.37, p = 0.017). The effect of condition remained significant (ß = -2.47; 95% CI [-2.95 -1.99]; Z = -10.12, p < 0.001). There was no significant interaction between Condition and Subjective_Memory (ß = 0.20; 95% CI [-0.23 0.62]; Z = 0.91, p = 0.36).

Additionally, we ran a model examining how Subjective memory is related to overall memory performance (Memory ~ Condition + SeenBefore + Subjective_Memory + Condition*Subjective_Memory + (Condition | Subject) + (1 | Video)). We found that now the effect of condition was no longer significant (ß = 0.24; 95% CI [-0.26 0.74]; Z = -0.93, p = 0.34). This meant that after including Subjective Memory into the model we did not observe a significant difference in overall memory accuracy across conditions. Subjective Memory was positively associated with better recall (ß = 2.31; 95% CI [1.95 2.68]; Z = 12.462, p < 0.001). No other factors were significant.

To summarise, a subjective memory score (a combination of vividness and confidence), was negatively associated with extension errors and positively associated with memory accuracy.

Experiment 2

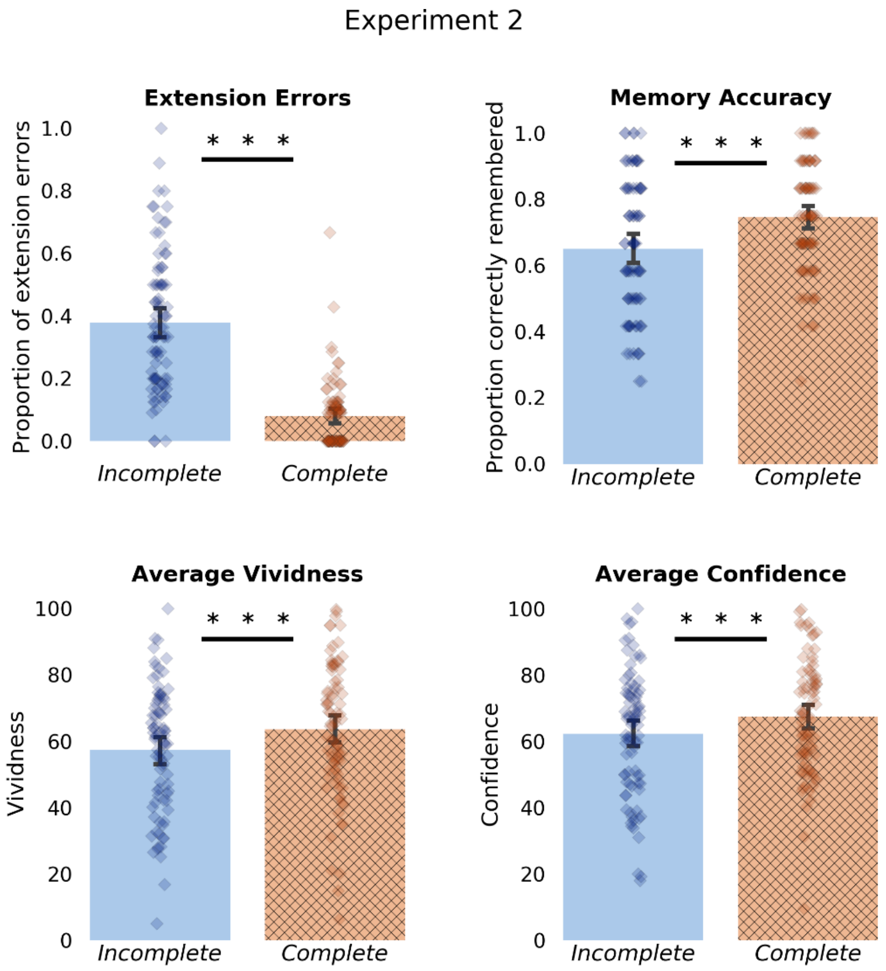

Supplementary Figure 4 **Experiment 2:** Top Panel: (Left) Replicating Experiment 1, participants were more likely to make an extension error in the *Incomplete* condition compared to the *Complete* condition. (Right) They also remembered more trials in *Complete* vs *Incomplete* condition. Bottom Panel: Participants provided higher vividness (Left) and confidence (Right) ratings in the *Complete* condition compared to the *Incomplete* condition. Error bars represent 95% confidence intervals of the mean. ***p<0.001.

First, we examined the correlation between vividness and confidence. Note in this experiment we did not have predictability ratings. We found that vividness and confidence were highly correlated ($r = 0.82$; 95% CI [0.80 0.83]; $t_{1423} = 53.43$, $p < 0.001$). Therefore, we again combined them into a single measure of Subjective_Memory, by averaging them together.

Next, we ran post-hoc analyses examining how Subjective_Memory was related to extension errors (Extension ~ Condition + SeenBefore + Condition*SubjectiveMemory + (1 | Subject) + (1 | Video)). We did not find a significant association between Subjective_Memory and extension errors (ß = -0.17; 95% CI [-0.40 0.06]; Z = -1.45, p = 0.15), nor did we find a significant interaction between Condition and Subjective_Memory (ß = -0.34; 95% CI [-0.73 0.04]; Z = -1.76, p = 0.08).

We also report the association between Subjective_Memory and Memory Accuracy. Similarly, to experiment 1, we found a positive association between Subjective_Memory and overall memory for the videos (ß = 2.26; 95% CI [1.96 2.57]; Z = 14.72, p < 0.001). We did not observe a significant interaction between Subjective Memory and Condition (ß = 0.21; 95% CI [-0.13 0.56]; Z = 1.20, p = 0.23).

Experiment 3

For experiment 3 we examined correlations between the continuous memory error measure (referred to as Memory_Error in the main manuscript) and vividness, confidence, and predictability. We note that in this experiment, participants provided vividness and confidence ratings after each recognition trial. In other experiments, subjective measures were collected before the recall trials.

We observed a strong positive correlation between vividness and confidence. Memory_Error was also correlated with confidence and vividness, but not with predictability. See Supplementary Table 5. Given the high correlation between vividness and confidence we again combined them into a single Subjective_Memory measure. We did not further explore predictability since it was not correlated with Memory_Error and showed weak correlations with the other measures.

When we ran a model examining the relationship between Memory_Error and Subjective Memory (Memory_Error ~ Condition + SeenBefore + Condition*Subjective_Memory + (1| Subject) + (1 | Video)). We found a positive association between Subjective Memory and Memory_Errors (ß = 0.36; 95% CI [0.14 0.58]; $t_{1158.89} = 3.28$, $p = 0.001$). Note, positive values of Memory_Errors are indicative of recognising more than the original presentation; negative meant that the participant recognised less, and zero means that the participant was perfectly accurate. We did not observe a significant interaction between Condition and Subjective_Memory (ß = 0.24; 95% CI [-0.06 0.54]; $t_{1163.49} = 1.56$, $p = 0.12$). The condition difference in Memory_Errors remained significant (ß = -0.95; 95% CI [-1.23 -0.66]; $t_{1127.22} = -6.58$, $p < 0.001$).

Table S5 Pairwise correlations between recognition memory and behavioural ratings for Experiment 3.

Variable 1	Variable 2	r	CI	$t\text{-stat}_{(1202)}$	p-value
Vividness	Confidence	0.76	[0.73, 0.78]	40.33	<0.001
Vividness	Predictability	0.16	[0.11, 0.22]	5.73	<0.001
Vividness	Memory_Error	0.08	[0.02, 0.13]	2.72	0.013
Confidence	Predictability	0.16	[0.10, 0.21]	5.52	<0.001
Confidence	Memory_Error	0.11	[0.05, 0.16]	3.77	<0.001
Memory_Error	Predictability	0.02	[-0.03, 0.08]	0.76	0.45

We also ran this model using the binarized error measure. This focused only on the positive Memory_Error scores (Bin_Memory ~ Condition + SeenBefore + Subjective_Memory*Condition + (1 | Subject) +(1 | Video)). We did not find a significant effect of Subjective_Memory (ß = -0.002; 95% CI [-0.21 0.21]; Z = -0.025, p = 0.98), or interaction between Condition and Subjective_Memory (ß = -0.04; 95% CI [-0.35 0.25]; Z = -0.3, p = 0.75).

Experiment 3 (Recognition Test)

Results including guess trials

These analyses are identical to the ones reported in the main text, except that we did not remove "guess" trials – where participants' response deviated more than ± 9 seconds from the encoded clip ending. We excluded one additional subject who made multiple responses that were deviating from the true encoding time, and they were outlier in the *Complete* condition.

Similarly to the main analyses, the (Memory_Error ~ Condition + SeenBefore + (Condition | Sub) + (1|Video)) model did not converge, and we dropped the random slope for condition. This model showed that participants showed better recognition performance for the *Incomplete* than the *Complete* video clips (-1.60 ± 5.18 vs -2.63 ± 5.50; $t_{1192.09} = -3.313$; $p < 0.001$) (see Supplementary Fig. 5).

We ran the post-hoc analyses where we transformed the continuous recognition responses into a binary extension measure. This analysis did show that participants were more likely to make extension errors in the *Incomplete* condition than the *Complete* condition (0.29 vs 0.20; Z = -3.5; $p < 0.001$) This is in line with what was reported in the main report.

Subjective ratings showed that *Incomplete* videos were rated as having a more predictable continuation than the *Complete* video clips (67.38 ± 27.5 vs 51.44 ± 31.40; $t_{51.46} = -8.78$; $p < 0.001$). However, unlike the analyses in the main report the vividness measure did not show any significant difference between the conditions (62.23 ± 27.89 vs 64.91 ± 26.84; $t_{51.97} = 1.74$, p = 0.08). Similarly, to the main report, there was no significant difference in confidence ratings (59.32 ± 29.77 vs 59.31 ± 27.91; $t_{52.17} = -0.01$ p = 0.98).

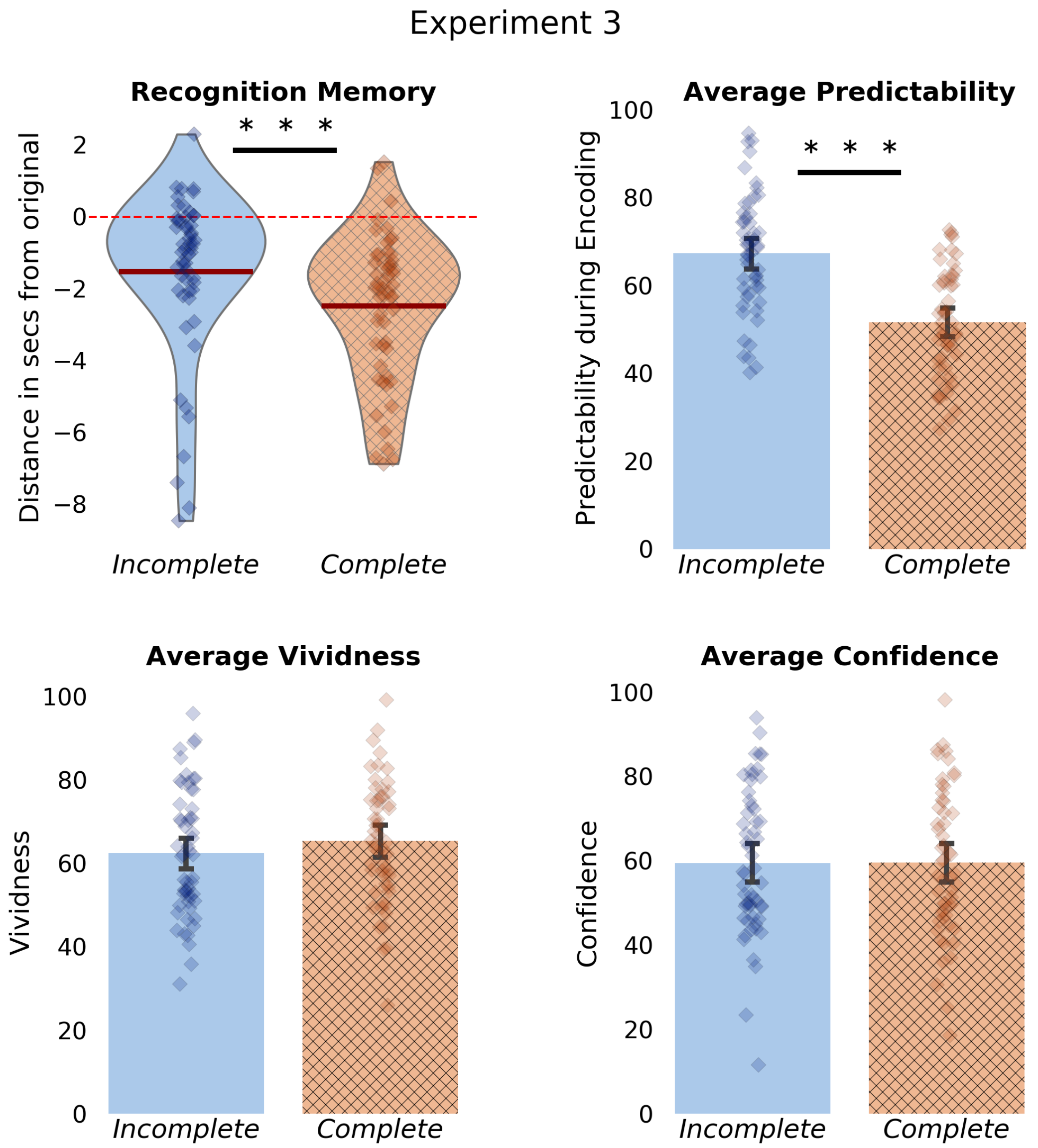

Supplementary Figure 5 **Experiment 3:** In the recognition task, participants underestimated the timing of the original video endings more in the *Complete* condition compared to the *Incomplete* condition. Replicating previous results, participants found the continuation of *Incomplete* video clips more predictable compared to *Complete* video clips. Vividness and confidence in memory did not defer significantly between the two conditions in this experiment.

Experiment 4

Similarly, to previous experiments we found a strong correlation between vividness and confidence (r = 0.79; 95% CI [0.77 0.81]; $t_{1468} = 49.56$, $p < 0.001$). We again combined them into a single measure of Subjective_Memory.

Fitting a logistic mixed effect model (Extension ~ Condition*Subjective_Memory + (1 | Subject)), showed that there wasn't a significant association between Subjective_Memory and extension errors (ß = -0.09; 95% CI [-0.29 0.11]; Z = -0.87, p = 0.38). We also did not find a significant interaction between Condition and Subjective_Memory (ß = -0.05; 95% CI [-0.41 0.30]; Z = -.29, p = 0.76).

We also fitted a logistic mixed effect model examining how omission errors are related to subjective memory (Omissions ~ Condition*Subjective_Memory + (Condition | Subject) + (1 | Video)). We found a significant effect of Subjective Memory (ß = 0.93; 95% CI [0.59 1.26]; Z = 5.48, $p < 0.001$). Interestingly, we also found a significant interaction between Subjective_Memory and Condition (ß = -0.27; 95% CI [-0.54 -0.02]; Z = -1.97, p = 0.048). The fitted relationship is shown in Supplementary Figure 6. As Subjective Memory increased there was a decrease in omission errors. This decrease was stronger for the *Updated* compared to the *Incomplete* condition.

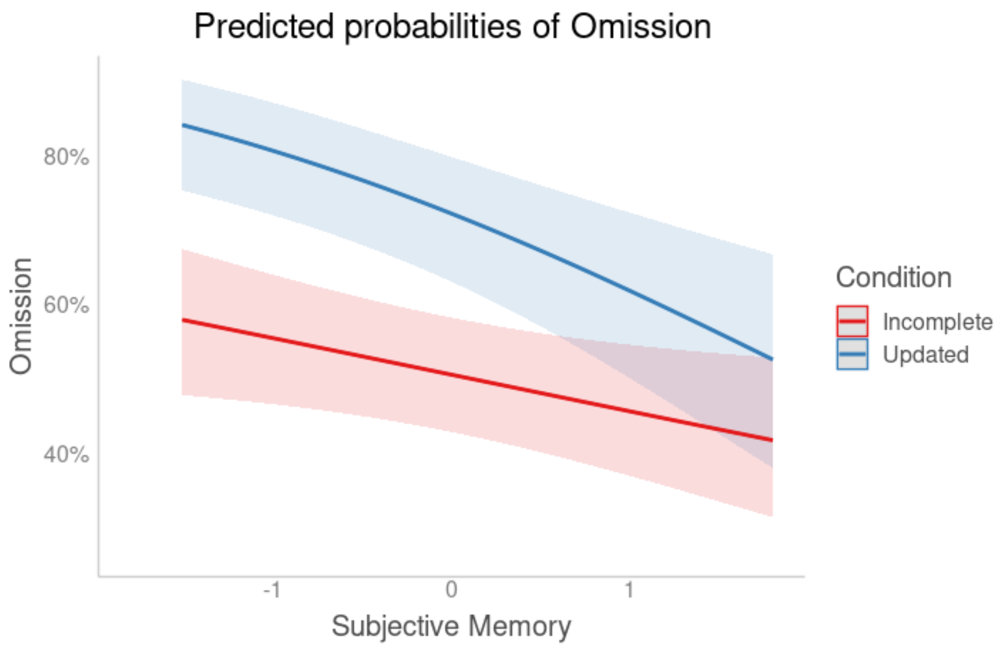

Supplementary Figure 6. **Experiment 4**: Predicted probabilities of making omission error in experiment 4 after a week delay. There was a significant Condition by Subjective memory interaction with steeper decline in omission errors for the *Updated* condition with increasing subjective memory.

Experiment 5a (Immediate)

We found a strong correlation between vividness and confidence (r = 0.86; 95% CI [0.84 0.88]; $t_{799} = 47.95$, $p < 0.001$). We again computed a single Subjective_Memory measure.

We then fit a logistic mixed effect model examining extension errors (Extension ~ Condition*Subjective_Memory + SeenBefore + (1 | Subject) + (1 | Condition)). We did not find a significant effect of Subjective_Memory (ß = -0.10; 95% CI [-0.52 0.30]; Z = -0.50, p = 0.61). We also did not observe a significant interaction between Subjective memory and Condition (ß = 0.1; 95% CI [-0.79 1]; Z = 0.23, p = 0.82).

Experiment 5b (Immediate)

We observed strong correlation between vividness and confidence (r = 0.73; 95% CI [0.70 0.75]; $t_{1262} = 37.77$, $p < 0.001$). We combined them into a single measure of Subjective_Memory.

Fitting a logistic mixed effect model (Extension ~ Condition*Subjective_Memory + (1 | Subject) + (1 | Video)), we found no significant association between Subjective_Memory and extensions (ß = -0.13; 95% CI [-0.45 0.18]; Z = -0.82, p = 0.41). We also did not observe an interaction between Condition and Subjective_Memory (ß = 1.1; 95% CI [-0.64 2.83]; Z = 1.23, p = 0.22).

We also used a logistic mixed effect model to examine the association between Subjective_Memory and omission errors (Omissions ~ Condition*Subjective_Memory + (Condition | Subject) + (1 | Video)). Similarly, to the delayed recall experiment 4, we found a significant association between Subjective_Memory and omission errors (ß = -0.45; 95% CI [-0.65 -0.26]; Z = -4.57, $p < 0.001$). We did not observe a significant interaction between Condition and Subjective_Memory (ß = -0.10; 95% CI [-0.36 0.15]; Z = -0.76, p = 0.44).

Subjective Memory overall summary

We did not observe a robust association across experiments between extension errors and subjective memory performance. Whenever, we observed association (experiments 1), we found that lower subjective memory was associated with higher probability of making an extension error. However, this relationship was not present in other experiments. We observed that higher subjective memory ratings were associated with less probability of making an omission error.

Experiment 6

We ran an additional experiment that aimed to examine whether the videos in experiments 4 and experiment 5b were perceived as more predictable when viewed in the *Incomplete* condition compared to the *Updated* condition. The procedure was identical to session 1 of experiment 4, except that participants were asked to provide predictability ratings for each video and did not complete a recall session. Participants watched the videos in a self-paced fashion and after each video they indicated how confidently they feel they can predict what would happen next. We recruited 40 (15 male, 25 female) participants using Prolific. Power calculations in simR based on results from experiment 1 showed that 40 participants provided us with 99% power to detect an effect of that was observed in experiment 1. Participants had a mean age of 28.26 (± 4.61).

Participants on average rated the *Incomplete* videos as more predictable compared to the *Updated* videos (66.9 vs 50.6 $t_{31.014} = -4.88$; Predictability ~ Condition + (Condition | Subject) + (Condition | Video); see Supplementary Fig. 7). These results are in line with previous findings from experiments 1 and 3 that videos that were cut just before an action was completed (*Incomplete* videos) were rated as having more predictable continuation compared to videos cut at the start of a new event.

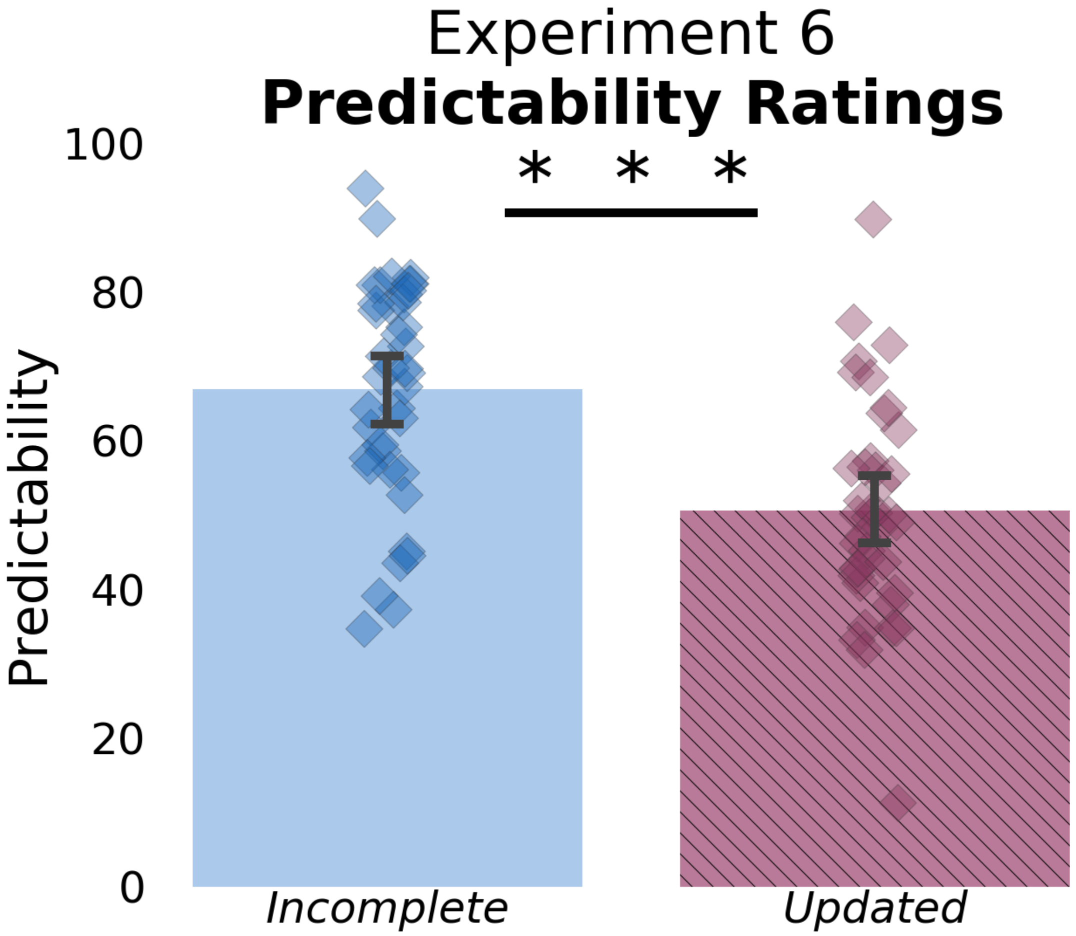

Supplementary Figure 7 **Experiment 6:** Predictability was higher for *Incomplete* videos compared to *Updated* clips. Predictability rating for everyday videos used in experiments 4 and experiment 5b. An independent group of participants rated how confidently they felt they could predict what would happen next after each video stopped. Barplot represents mean and 95% CI. Each marker represents the average predictability rating within a condition for a single subject.

References

Makowski, D., Ben-Shachar, M. S., Patil, I., & Lüdecke, D. (2020). Methods and algorithms for correlation analysis in R. *Journal of Open Source Software*, *5*(51), 2306.
